# Economical and Versatile Subunit Design Principles for Self-Assembled DNA Origami Structures

**DOI:** 10.1021/acsnano.5c06681

**Published:** 2025-08-19

**Authors:** Wei-Shao Wei, Thomas E. Videbæk, Daichi Hayakawa, Rupam Saha, Juanita Pombo, Gaurav Arya, W. Benjamin Rogers, Seth Fraden

**Affiliations:** † Martin A. Fisher School of Physics, 8244 Brandeis University, Waltham, Massachusetts 02453, United States; ‡ Materials Research Science and Engineering Center (MRSEC), Brandeis University, Waltham, Massachusetts 02453, United States; § Thomas Lord Department of Mechanical Engineering and Materials Science, 3065 Duke University, Durham, North Carolina 27708, United States

**Keywords:** self-assembly, programmable assembly, patchy colloids, DNA nanotechnology, DNA origami, cryo-EM, multi-body refinement

## Abstract

We describe a modular design approach for creating versatile DNA origami subunits that can target diverse self-assembled structures. The subunit consists of a constant “core module” with variable “bond modules” and “angle modules” added to its exterior, controlling interaction specificity, strength, and structural geometry. The design features flexible joints between subunits, implemented by using single-stranded angle modules, whose mechanical properties and possible conformations are characterized by cryogenic electron microscopy and coarse-grained molecular modeling. We demonstrate the design’s versatility through the assembly of structures with different Gaussian curvature, including sheets, spherical shells, and tubes. Our findings suggest that incorporating a judicious amount of flexibility in the bonds provides error tolerance in design and fabrication while maintaining target fidelity. Furthermore, off-target assemblies potentially introduced by flexibility can be counterbalanced by increasing the number of distinct bonds. This approach enables precise targeting of specific structural binding angles across a broad range of configurations by eliminating unfavorable interactions.

## Graphical abstract

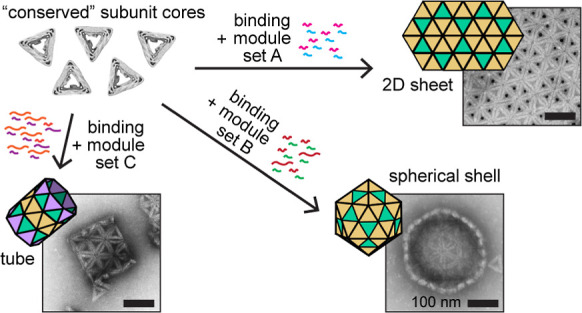

Self-assembly of elementary nanoscale subunits into complex supramolecular structures is a hallmark of living systems.^1–7^ Exploiting this approach to create new synthetic materials has been a longtime goal of bioinspired material science.^8–12^ In this pursuit, the soft-matter field has identified attributes for idealized self-assembling subunits, colloquially referred to as “patchy particles”.^13–18^ An ideal patchy particle is one in which there is arbitrary control over the particle’s shape and size, as well as control over interparticle interactions including bond valency, angle, specificity, and strength (Figure 1A). While computer simulators can readily create ideal patchy particles, synthesizing them in the lab has been challenging.

**Figure 1:**
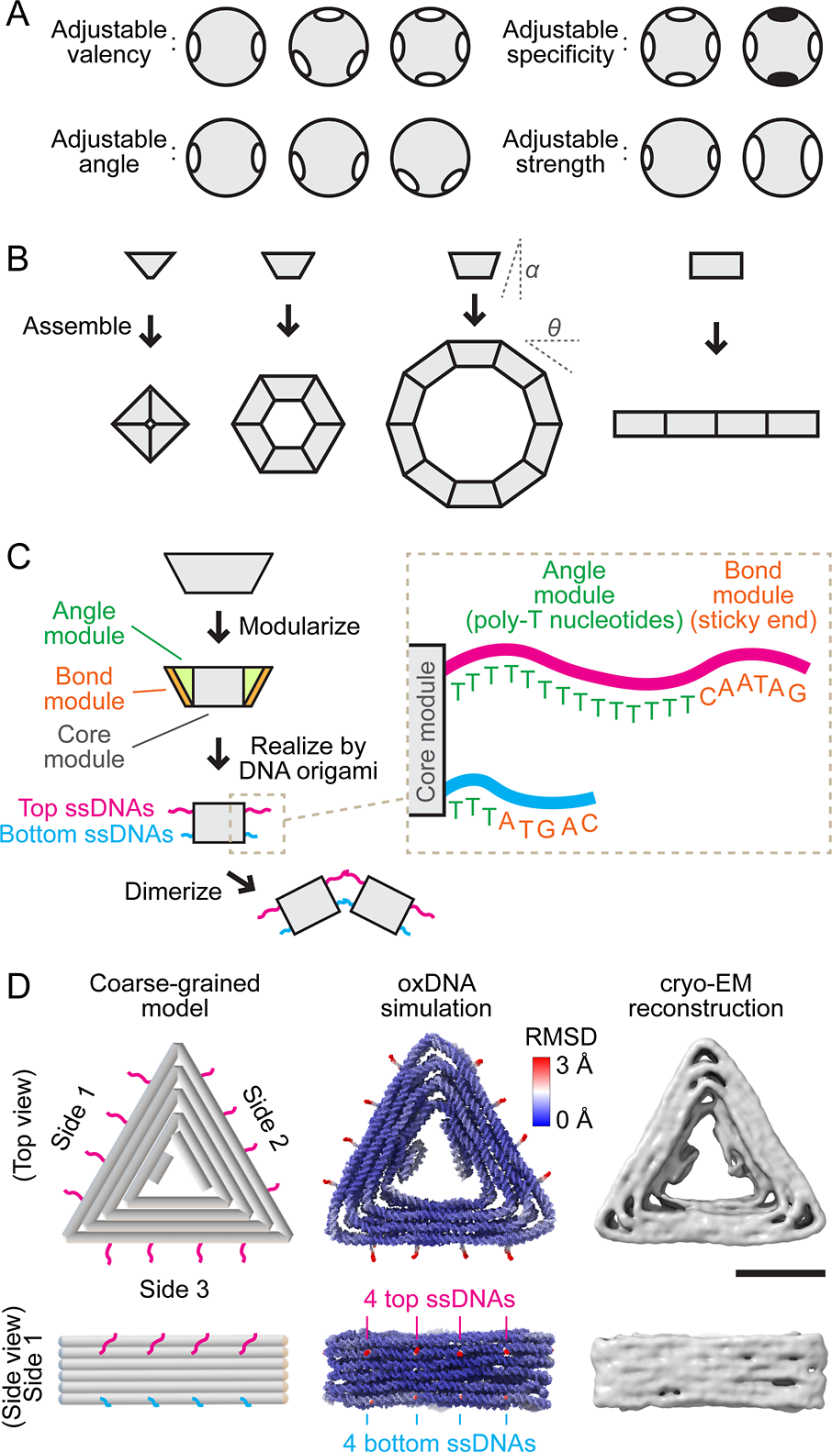
Design principles of modular subunits. (A) Schematic illustrating adjustable attributes of patchy particles—valency (upper left), bond angle (lower left), bond specificity (upper right), and bond strength (lower right). The white/black patches on particles represent binding sites, with like-colors binding to each other and no interaction between unlike-colors. (B) Subunits with a designed bevel angle α specify the binding angle θ = 2α. (C) Illustration of applying modularity to a subunit block, dividing it into a conserved core module and variable angle and bond modules. On each core face, the variable modules are made of four top (pink) and four bottom (cyan) ssDNA strands. The angle module consists of ssDNA poly-T segments (green sequences); the bond module consists of ssDNA sticky end segments (orange sequences). (D) Left: schematic of the subunit; the variable modules made of ssDNAs are also sketched. Each cylinder represents a dsDNA helix. Middle: coarse-grained oxDNA simulated subunit with color-coded structural rigidity. The core module is mostly rigid (blue); the angle and bond modules are very flexible (red). Right: cryo-EM reconstructed subunit core module. Scale bar: 20 nm.

In the last several years, we succeeded to manufacture colloids with the ideal attributes of patchy particles from DNA origami and demonstrated self-assembly into capsid shells^19,20^ and tubules.^21^ The bond angle was controlled by beveling the subunit edges, thereby specifying the local curvature of self-closing assemblies (Figure 1B). Bond strength and selectivity were achieved through complementary lock-and-key domains placed on the sides of the triangular blocks. While effective, this approach requires encoding both bond angle and interaction specificity directly into each subunit’s geometry. As a result, designing new subunits with different bond angles or specificities requires scaffold rerouting resulting in the need to replace all of the DNA origami staples, which is costly, labor-intensive, and demands specialized expertise.

Here, we employ a simple and economical modular design principle. Modularity—widely used in engineering—divides a system into smaller components that can be independently created, modified, and exchanged, offering greater flexibility in design, cost, and function. This concept has been successfully applied in DNA nanotechnology to create subunits with diverse geometries and interactions for both two- and three-dimensional assemblies.^22–27^ In our approach, each subunit is divided into a universal “core module” and variable “angle” and “bond” modules, which encode local curvature and interaction specificity, respectively (Figure 1C).

As before, we fabricate subunits using DNA origami,^22,28^ with a triangular core module having a maximum valency of three. On each of the three core faces, eight single-stranded DNA (ssDNA) oligos extend, containing the angle and bond modules. The angle modules, composed of polythymidine (poly-T) nucleotides, act as spacers to control local curvature, while the bond modules program binding specificity and strength through their base sequences (Figure 1C,D). Interactions can be selectively deactivated by omitting the corresponding ssDNA extensions.

Crucially, the designs feature flexible joints between pairs of subunits, in large part because of the use of ssDNA in the angle module. Using cryogenic electron microscopy (cryo-EM) and multi-body refinement, we quantify the bond-angle distribution in subunit pairs. This technique was initially developed to reconstruct molecular motion of proteins with multiple flexible complexes.^29,30^ Conventional wisdom leads one to avoid flexibility in self-assembly due to the lack of precision. However, we find that flexible joints offer several advantages. First, flexible joints lead to an increased error toleration in the design and fabrication of subunits. Second, the large flexibility facilitates two subunits coming together in a configuration that results in a bond, increasing the on-rate. When the bond flexibility is large enough to cause undesirable formation of off-target structures, selectivity of the target structure can be re-established by increasing the number of distinct bond modules.^31–34^

Using this modular design with flexible joints, we demonstrate the generation of a rich set of distinct subunits. Once the core design is established, each subunit variant requires at most 12% oligo modifications compared to redesigning from scratch, with a corresponding reduction in the time to design and the cost of variant oligos by roughly a factor of 8. This design principle not only significantly reduces the design and synthesis effort for variants but also makes the platform readily accessible to researchers without expertise in DNA origami design. The core, which contains all of the origami-designed portions, remains invariant, while the variable angle and bond modules are simple to design. Here, we showcase the design and fabrication of subunits that self-assemble into various structures, exemplified by ones with zero-Gaussian curvature (2D tilings and short cylindrical tubes) and positive-Gaussian curvature (hollow spherical shells of varying icosahedral symmetries).

## Results and Discussion

The subunit we employ is a rigid three-dimensional right equilateral triangular prism made from DNA origami, with a rectangular cross-section of 15 × 10 nm, formed by construction of a 6 × 4 square lattice of double-stranded DNA (dsDNA) helices and an edge length of 52 nm (Figure 1D). The triangular shape is chosen for its mechanical stability, resisting shear in the plane and topologically suppressing net twist along the perimeter due to the closed-loop origami structure. Rigidity is enforced by using a thick cross-section. All information necessary to self-assemble these subunits into user-prescribed higher-order structures is encoded in the variable angle and bond modules. While the core module fabrication is similar to our prior work,^19–21^ we employ different design principles for the angle and bond modules, as detailed below.

### Design Principle and Fabrication of Subunits

Subunit geometries and interactions guide the size and shape of the structures they form (Figure 1B).^19–21,24,35–39^ To reduce the burden of redesigning subunits for different targets,^19–21,34^ we introduce a design principle that decouples geometry and interactions from the subunit body (Figure 1C). A universal core module is fabricated as a pegboard. Along each outer face of the core, replaceable angle and bond modules act as pegs that encode geometry and binding information to personalize the properties of each subunit.

The core module is made from DNA origami, and extruding from each of the three triangle core faces are four “top” and four “bottom” ssDNA strands (Figure 1C,D). Each strand consists of three segments. The innermost is an invariant anchor segment, containing a specific DNA sequence, that strongly hybridizes inside the core (Figures S1A and S2). The next two overhanging segments extend out from the core. They are called the angle and bond modules and vary with each design (Figure 1C right). The angle module, closest to the core, comprises a series of poly-T nucleotides with varying lengths, determining the binding angle between subunits (θ in Figure 1B). Next comes the bond module, consisting of 5–7 unpaired nucleotides, referred to as a “sticky end”, which binds specifically with a complementary bond module on another subunit via hybridization. A custom sequence optimization procedure^34,40,41^ is employed to minimize cross-talk between nonpaired segments (Methods). The organization of these 8 ssDNA strands ensures correct subunit–subunit binding orientation without offset. Consult Methods and Figures S1–S4 for details.

To achieve effective assembly, intersubunit binding strengths must balance the requirement of the thermodynamic stability of the target structure with the ability to anneal kinetic traps.^20,42–45^ This critical balance is achieved by fine-tuning the bond module hybridization strength through adjustable parameters such as the number of ssDNA strands per face, the nucleotide count in each bond module, the solution ionic strength, and the temperature. As a rule of thumb, we aim to maximize both the on- and off-rates subject to constraints. We maximize the on-rate by using the highest practical monomer concentration, with limits imposed by cost and the need to avoid nonspecific aggregation which, for our colloidal DNA origami system, is about 5 nM. At the same time, we maximize the off-rate to accelerate equilibration by choosing the weakest binding strength that still allows complete assembly. This approach allows subunits to quickly dissociate from incorrect or partial assemblies, thereby preventing kinetic traps and promoting the formation of correctly assembled structures. See Methods for details.

### Self-Assembled 2D Tilings: Program Binding Specificity Using Subunits with Conserved Core

We first demonstrate the versatility of our modular design by creating two-dimensional (2D) structures with different tiling patterns, achieved by varying the interaction specificity encoded in the bond modules (Figure 1C and 2 second column). For 2D assemblies, the binding angle θ between adjacent subunits is zero degrees. This is accomplished by setting all 8 ssDNA strands of the angle module to the same length, e.g., three poly-T (Figure 2A,B first column). Note that when extruding ssDNA from a dsDNA origami structure, it is a common practice in DNA origami design to insert a short length of poly-T base to overcome steric hindrances within the DNA origami core and to relieve stress.^46–48^

**Figure 2:**
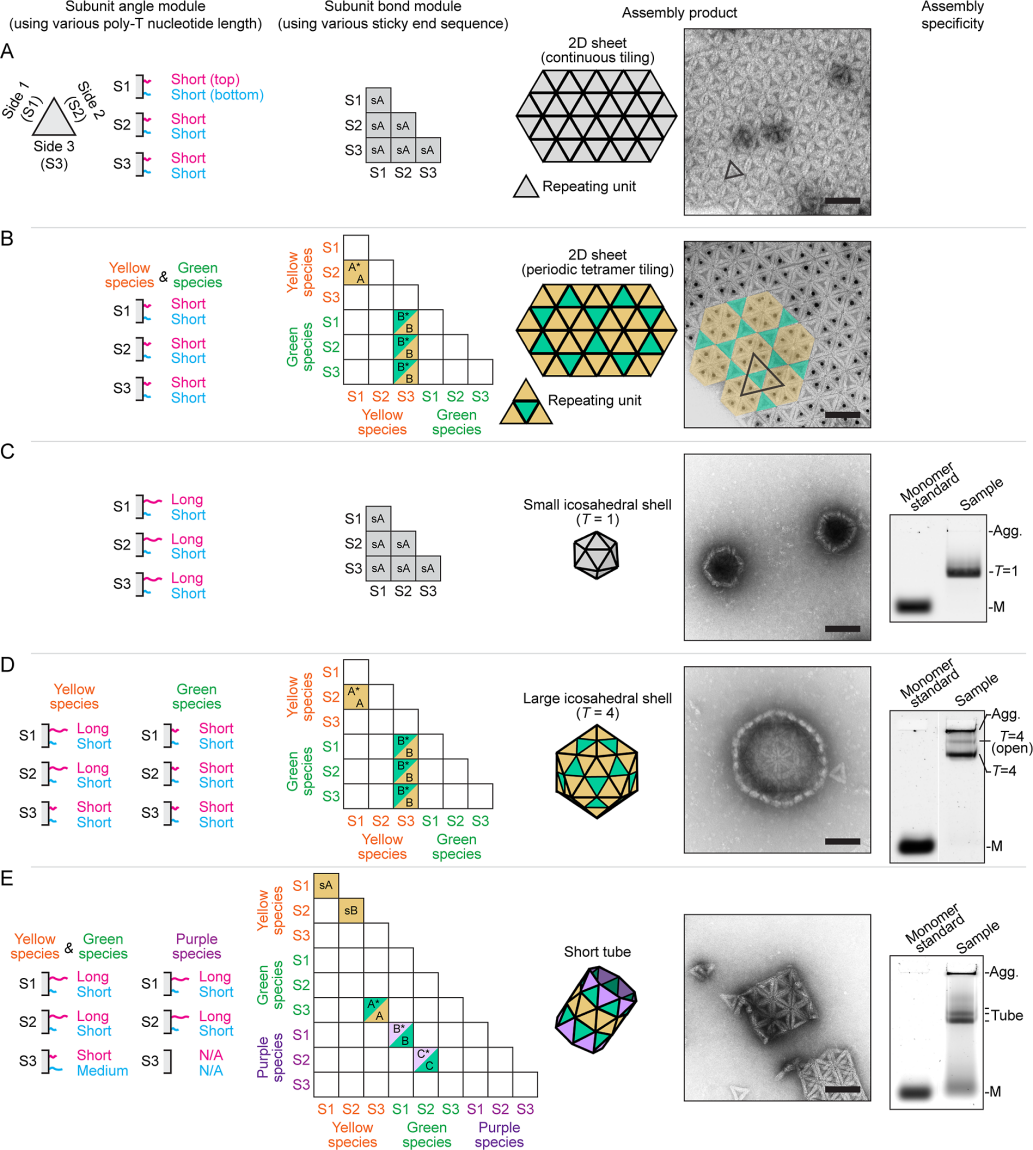
Structures self-assembled from subunits with the same conserved core. (A–E) Subunits deploying the same core module, but different angle and bond modules self-assemble into (A) planar sheets with continuous tiling, (B) planar sheets with hexagonal tiling, (C) *T* = 1 capsids, (D) *T* = 4 capsids, and (E) short tubes. A schematic illustrating the angle modules is provided. Short, medium, and long ssDNA contains a 3, 8, and 14 poly-T segment, respectively (1st column). The interaction matrix encoded into the bond modules is also stated. Matrix elements with a single color or two colors represent a bond between identical species or between two different species. The black letters indicate distinct bond sequences detailed in Methods (2nd column). The sketches and TEM images show the final assembled structures. Scale bar: 100 nm. The yellow-coded subunits in (B) are labeled with gold nanoparticles to be distinguishable under TEM (3rd column). The gel electrophoresis reveals high target yield and specificity as detailed in Figures S6 and S7. “Agg.” and “M” indicate aggregate and monomer population, respectively (4th column).

The simplest interaction matrix uses 3-fold symmetry, where each of the three subunit edges (S1, S2, S3) have identical bond module sequences, allowing pairing between any two edges (Figure 2A second column, Figure S4B). This leads to a 2D sheet with continuous tiling without orientational order using a single triangle as the fundamental building block (Figure 2A third column; see Methods for the detailed design).

More complex tiling patterns can be created by assigning specific interaction rules between subunits.^49^ We demonstrate this by using two species of equilateral triangular subunits (with a 3:1 number ratio) to design a hexagonal tiling pattern with a tetrameric repeating unit (Figure 2B, see Methods for detailed design). The two subunit species share the same core and angle modules, differing only in their bond modules (Figure 2B second column, Figure S4B).

### Self-Assembled 3D Structures: Program Local Curvature Using Subunits with Conserved Core

We next demonstrate the ability to assemble three-dimensional (3D) structures with different Gaussian curvature by varying the angle modules. The binding angle between two subunits is controlled by the length differences between the four top ssDNA strands and the four bottom ssDNA strands on each face (Figure 1C,D). We vary the number of poly-T nucleotides in the angle module (*l*_top_ and *l*_bottom_) while keeping the same base number in the bond module. Our hypothesis was that a zero degree binding angle would form when *l*_top_ = *l*_bottom_, a positive angle when *l*_top_ > *l*_bottom_, and a negative angle when *l*_top_ < *l*_bottom_. These binding angles control the local curvature. We target the creation of icosahedral shells (capsids) and cylinders following the Caspar–Klug theory^2^ to transform 2D triangle tilings into 3D structures.

For the icosahedron, we assemble 20 equilateral triangles with an interaction matrix identical to the one-species planar 2D sheet (Figure 2A,C second column, Figure S4B). However, we program a positive binding angle of 41.8° by setting *l*_top_ = 14 poly-T and *l*_bottom_ = 3 poly-T (Figure 2C first column). The value of *l*_top_ was chosen through a systematic exploration of different values of *l*_top_ (see the guidance in Figure S5A). The resulting *T* = 1 capsid, comprising 20 identical subunits, was assessed using transmission electron microscopy (TEM) and gel electrophoresis (Figure 2C third and fourth column, Methods). Note, the triangulation number, *T*, specifies the minimum number of distinct local symmetries/interactions required to form the corresponding capsid.^2^ The design exhibits high target specificity with no experimentally identifiable byproducts, achieving an overall yield of 62%, meaning this percentage of input subunits assembles into the target *T* = 1 shell, while the remainder forms small oligomers or aggregates (Figure 2C fourth column, Figures S6A and S7A).

We then create *T* = 4 icosahedral shells using the same interaction matrix as the two-species planar 2D sheet (Figure 2B,D second column, and Figure S4B). Each *T* = 4 capsid comprises 20 planar triangular-shaped tetramers (80 total subunits) and employs two subunit species (Figure 2D). Within the flat tetrameric triangle, *l*_top_ = *l*_bottom_ = 3 poly-T is designed for a 0° binding angle along the 3-fold (yellow-green) bond; between adjacent tetrameric triangles, *l*_top_ = 14 poly-T > *l*_bottom_ = 3 poly-T is set for a 41.8° structural binding angle along the 5-fold (yellow–yellow) bond (Figure 2D first column). This *T* = 4 structure demonstrates the modularity of our approach, utilizing two distinct angle modules (0° and 41.8°) and four distinct bond modules (A, A*, B, and B*) (Methods, Figures S3 and S4). TEM and gel electrophoresis show an overall yield of 34% with high target specificity and no experimentally identifiable byproducts (Figure 2D fourth column, Figures S6B and S7B,C). Kinetic studies further represent how the reaction evolves over the whole assembly period (Figure S8).

To further validate that our design principle is viable for a variety of complex structures, we assemble short cylindrical tubes, or “tubelets” (Figure 2E). Cylinders have zero Gaussian curvature, requiring both positive and negative binding angles in a single subunit if built from equilateral triangles.^21^ We design all component subunits with two bonds having a positive binding angle (*l*_top_ = 14 poly-T > *l*_bottom_ = 3 poly-T) along the circumferential direction and one bond with a negative angle (*l*_top_ = 3 poly-T < *l*_bottom_ = 8 poly-T) in the direction parallel to the axis of symmetry (Figure 2E first column). An interaction matrix with translational symmetry is used (Figure 2E second column, Methods). The resulting cylinders are monodisperse in length but variable in diameter due to the large flexibility between subunits.^50–52^ The achiral tubes are classified by their lattice number (*m*, 0), where *m* indicates the number of subunits in the shortest self-closing loop along the triangular lattice. Gel electrophoresis shows yields of 20%, 12%, and 5% for (6, 0), (7, 0), and (8, 0) tubelets, respectively (Figure 2E fourth column, Figures S6C, S7D–E, and S9). This polymorphism can be minimized by increasing the number of distinct bonds in the tubelets, ensuring that only one structure with the desired curvature can close while alternative configurations are prevented from forming, as demonstrated in our previous work.^31,34^

### Characterizing the Mechanical Properties of Joints between Adjacent Subunits

While the core of the triangular subunit is very rigid, the joints between subunits exhibit flexibility, with thermal fluctuations causing the binding angle between two bound subunits to vary over a broad range (Figure 3A top sketch). This flexibility arises from the use of ssDNA strands in the bonds, particularly in the variable angle modules (which contain 3–14 bases of single-stranded poly-T) (Figure 1C). The short persistence length of ssDNA^53^ leads to coiling of long strands, creating spring-like joints that bend, twist, and stretch.

**Figure 3:**
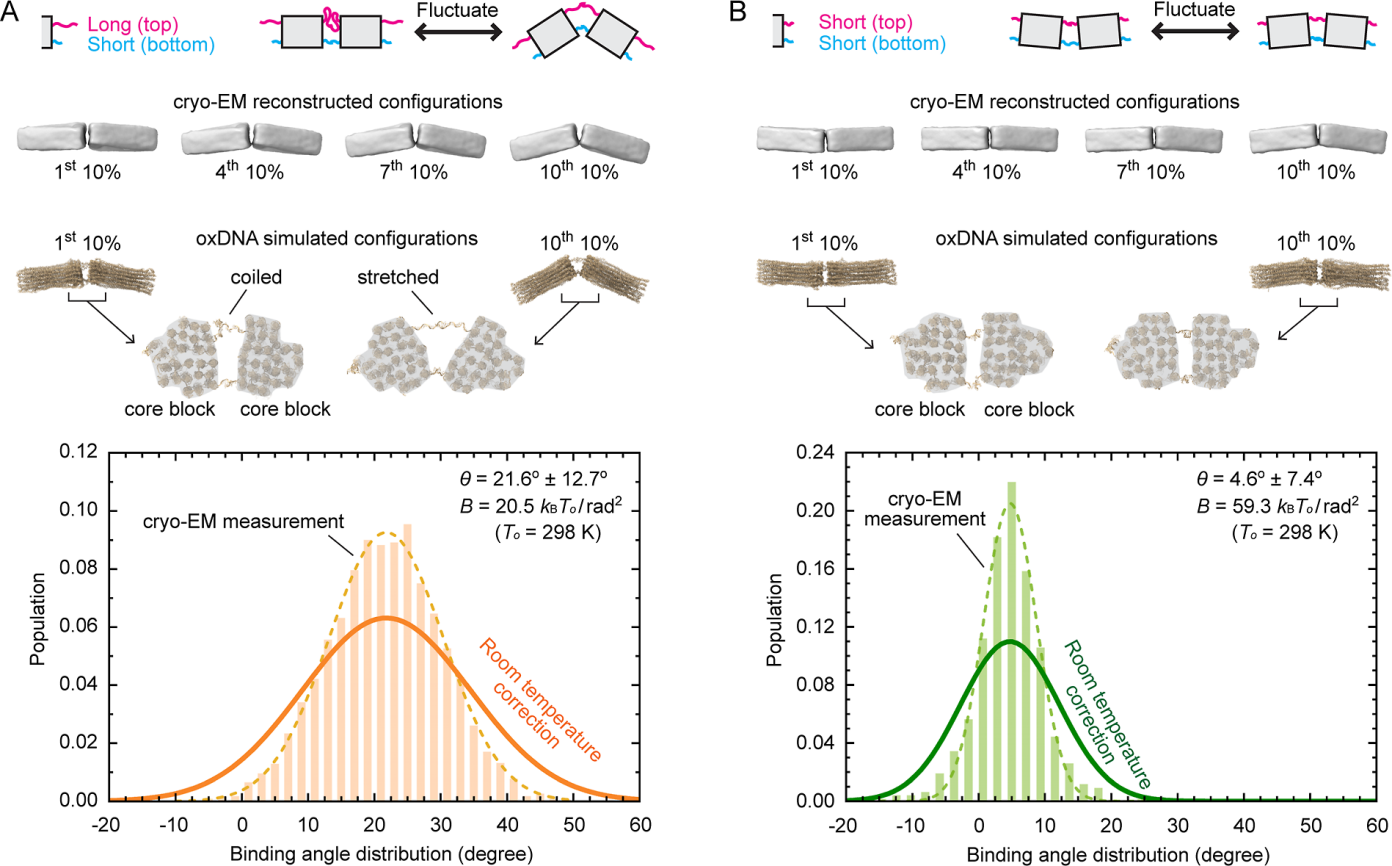
Angular distribution between subunits characterized by cryo-EM and oxDNA simulations. (A) The dimerized subunit pair with angle modules *l*_top_ = 14 poly-T > *l*_bottom_ = 3 poly-T (used in 5-fold bonds of Figure 2C,D) has a broad angular distribution. The four cryo-EM reconstructions represent average configurations of the most flattened 10% dimer ensemble, the most bent 10% dimer ensemble, and another two states in between (top). The corresponding oxDNA simulations illustrate the idea that the long ssDNA (*l*_top_ = 14 poly-T) acts as a Brownian spring and the short ssDNA (*l*_bottom_ = 3 poly-T) acts as a hinge (middle). The binding angle distribution of the joint is extracted directly from cryo-EM observation (bar graph, 136 K), fitted by a Gaussian (dashed curve, 136 K), and rescaled to room temperature (solid curve, *T*_0_ = 298 K) (bottom). (B) The dimer joined by angle modules *l*_top_ = *l*_bottom_ = 3 poly-T is more rigid compared to (A), visualized by smaller changes in configurations (top, middle) and a narrower binding angle distribution (bottom).

To quantify this flexibility, we employ cryo-EM along with multi-body refinement^29^ to measure the distribution of binding angles in a dimer (Figure 3). We focus on dimers as they represent the fundamental assembly step and are the simplest to study experimentally. Because the origami core module is designed to be much more rigid than the joint (the angle and bond modules), we approximate the molecular motion as two rigid subunit cores with varying relative orientations. We complement these experiments with molecular dynamics simulations using the oxDNA coarse-grained model^54^ to elucidate ssDNA bonding strand conformations and compare with our experimental measurements (Methods, Figure S10).

Multi-body refinement analysis determines the relative orientation of the two cores for each dimer image, enabling decomposition into bend, twist, and stretching modes through principal component (PC) analysis.^55–57^ See Methods for some technical details, while a full description of the method is presented in another publication with modeling.^58^

To obtain the room-temperature distribution of the dimer angles, temperature corrections are required. Cryo-EM involves cooling the sample from room temperature to the vitrification temperature of water, assumed to be 136 K, in about 10^–4^ seconds.^59–64^ The measured angular distribution fits well to a Gaussian, characterized by an average angle and standard deviation σ, implying that the bond acts as a spring. We assume the angular distribution equilibrates to 136 K during vitrification because the rotational diffusion time constant of a free monomer is of the order of 10^–9^ s.^65^ Assuming that none of the physical properties of the bond, including its spring constant, are temperature dependent,^66^ the angular distribution at room temperature will therefore be Gaussian with the same average angle but with a rescaled standard deviation following the relation σ^2^_298Κ_/σ^2^_136Κ_ = 298 Κ/136 Κ. While this approach offers a first-order estimate of the angular distribution at room temperature, it does not capture the full complexity of the system. Preliminary simulations indicate that the bond’s elastic properties deviate from both purely entropic and temperature-independent models, suggesting additional factors are involved (Methods, Figure S10D). Further investigation is needed to fully understand these effects.

Cryo-EM images (Figure 3A top) and oxDNA simulations (Figure 3A middle, Figure S10A) underpin our physical intuition regarding the long ssDNA (*l*_top_ = 14 poly-T) acting as a spring and short ssDNA (*l*_bottom_ = 3 poly-T) as a hinge. The relative angle between subunits determined from cryo-EM exhibits a Gaussian distribution with an average binding angle of 21.6° and a standard deviation of 12.7° after temperature rescaling (Figure 3A bottom, Figure S11, Video S2). An estimated room-temperature bending elastic modulus of 20.5 *k*_B_ *T*_0_/rad^2^ (*T*_0_ = 298 K) of the joint is extracted from the distribution of binding angles. In contrast, joints with all-short ssDNA strands (*l*_top_ = *l*_bottom_ = 3 poly-T) are more rigid, exhibiting a narrower Gaussian distribution with a standard deviation of 7.4° and an average binding angle of 4.6° at room temperature (Figures 3B, S10B, S11, and Video S1). This corresponds to a larger room-temperature bending elastic modulus of 59.3 *k*_B_ *T*_0_/rad^2^. Besides the dominant bending motion, we observe minor twisting and stretching modes, revealing that it is oversimplified to view the bottom row of ssDNA as a hinge with only one degree of freedom (Figure S12).

Lastly, comparing oxDNA simulations with experimental results, we find that the simulated angle distributions qualitatively agree with the cryo-EM results but are somewhat narrower and the average binding angles also differ slightly (Figure S10). This discrepancy may arise from experimental distributions averaging over numerous folded origami structures, including potential misfolding instances and missing staples, while simulations use a single preassembled, defect-free dimer (Methods). Additionally, the highly coarse-grained treatment of DNA and counterions in the oxDNA model, especially at the subunit interfaces, may restrict hinge relaxation, resulting in a more rigid bond, as previously reported.^67^ These discrepancies between simulations and experiments in binding angle distributions warrant further investigation.

### Flexible Joints between Subunits as a Feature

The mechanical properties of flexible subunit–subunit joints influence larger-scale assemblies. The angle module with *l*_top_ = 14 poly-T > *l*_bottom_ = 3 poly-T enables the formation of 5-fold vertices in both *T* = 1 and *T* = 4 capsid assemblies, with structural binding angles of 41.8° as demonstrated in Figure 2C,D. Interestingly, the cryo-EM measurements show an average dimer angle of only 21.6°; however, the required 41.8° angle can still form due to the joint’s flexibility, as shown in Figures 3A and 4A. It is noteworthy that this amount of flexibility is insufficient to allow other closed forms (e.g., octahedra with 70.7° binding angles), explaining the high assembly specificity and yield (Figures S6 and S7).

**Figure 4:**
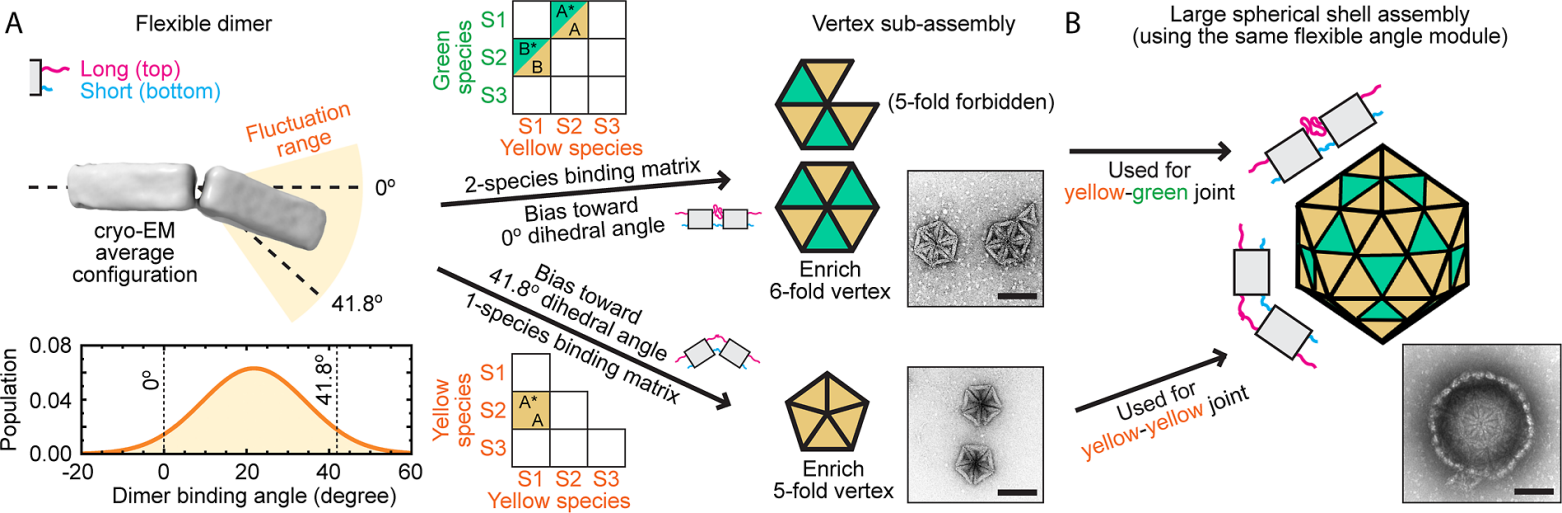
Flexible subunit–subunit joints allow formation of bonds with different binding angles. (A) By assigning an appropriate interaction rule via bond modules, subassembly of a 5-fold vertex (bottom right, corresponding to a 41.8° binding angle) or a 6-fold vertex (top right, corresponding to a 0° binding angle) can be preferentially enriched using subunit–subunit joints with the same flexible angle module (left, characterized in Figure 3A). (B) Following the same principle, a *T* = 4 large capsid shell can be constructed using the same angle module for all its joints to target the required 0° and 41.8° binding angles. The *T* = 4 design encodes the same interaction matrix, as shown in Figure 2D. Scale bar: 100 nm.

A second noteworthy point to consider is that while the fluctuating dimer can reach 0° (Figures 3A and 4A), we never observe planar structures, likely because closed icosahedra are energetically and kinetically favored over large open sheets. Considering the relatively low energy cost of bending, closed structures are energetically favorable over open structures with the same number of subunits due to the elimination of edge tensions.^68–71^ Additionally, all subunits in an icosahedron have three neighbors, while subunits along the edge of an open sheet have fewer than three neighbors, causing open structures to dissociate more readily than closed ones.

Our results indicate that flexible joints enhance assembly fidelity provided that two key design criteria are satisfied. First, the allowable range of angle fluctuations between paired subunits should encompass the intended (target) binding angle; this is an energetic condition. Second, a kinetic condition arises when flexibility permits multiple closed-loop structures. While any closed structure is stable because each subunit forms three bonds, the structure with the fewest subunits likely assembles most rapidly and is kinetically favored (Figure S13). Fewer subunits correspond to a smaller radius of curvature and a larger binding angle; hence, among the possible closed structures allowed by the angle fluctuation range, the smallest structure with the largest binding angle preferentially forms.^48,52^ These design principles provide greater error tolerance in both design and fabrication, allowing target structures to form reliably, even with small variations in poly-T segment length (Figure S9). As a result, successful assembly could be achieved without precise bond geometry optimization. Additionally, the flexible joint design can yield faster assembly rates and higher yields than previous designs requiring exact subunit angles,^19,72^ which are limited by higher entropic penalties (Figure S8B).

If excessive flexibility leads to polymorphic, off-target structures, specificity and target structure yield can be restored by increasing the number of distinct subunits, effectively balancing economy and complexity.^31–34^ Specifically, the angle module with *l*_top_ = 14 poly-T > *l*_bottom_ = 3 poly-T (Figure 3A) can produce structures with different binding angles depending on the number of distinct subunits (Figure 4). In Figure 4A, we perform experiments with one side of the triangle passivated and an interaction matrix designed so that only structures with a single vertex can form. When only one subunit species is used, allowing any two subunits to bond, flexible joints favor the formation of 5-fold vertices with 41.8° binding angles (Figure 4A bottom right). In contrast, requiring two distinct subunit species to form dimers restricts assembly to substructures containing only even numbers of monomers, making structures with 5-fold symmetry impossible and thus favoring 6-fold vertices with 0° binding angles (Figure 4A top right). By adjustment of the interaction matrix, different vertices and local structural curvatures can be selectively enriched from subunits with the same angle module (Figure S14A).

Finally, we demonstrate that flexible bonds can effectively target assemblies of higher-order structures by leveraging specificity in bond modules to achieve a high specificity despite substantial angular flexibility. We illustrate this principle by constructing a *T* = 4 icosahedral shell using a single, highly flexible angle module (*l*_top_ = 14 poly-T > *l*_bottom_ = 3 poly-T; Figure 4B) for all joints. Although this angle module can accommodate both required binding angles of 0° and 41.8° (represented as yellow-green and yellow–yellow bonds, respectively, in Figure 4), specificity introduced through distinct bond modules successfully suppresses the formation of off-target structures, resulting in high product specificity without experimentally identifiable byproducts (Figure S14B). While utilizing two tailored angle modules matched specifically to each target angle achieved a higher yield (34%; Figure 2D) compared with a single flexible module (14%), our primary aim here was to demonstrate that increased flexibility allows a range of specific binding angles to be selected by increasing the number of distinct bond modules.

## Conclusions

We applied the engineering notion of modularity to nanoscale subunits, enabling the construction of user-prescribed large and complex self-assembled structures in an economical manner. In this design strategy, each subunit contains one right triangular prism core module and a set of angle and bond modules, comprising 8 overhanging ssDNA strands, located on each of the three core faces. The angle and bond modules are largely independently tunable, controlling the binding angle and the binding strength/specificity, respectively, while the core module is a nonfunctional block conserved among all variants.

This modular principle dramatically reduces the design and synthesis effort compared to prior approaches,^19–21,34,37^ as only 24 out of 204 strands in a single subunit need to be redesigned for each variant. Designs of the angle and bond modules rely on simple geometric and hybridization rules. The former contains poly-T segments; the only design consideration is their length (Figure S1). The latter uniquely pairs with a second bond module on another subunit; the most important design consideration is its complementary hybridization sequences (Figures S1A,B and S3). Our bond module design and assembly conditions also optimize the kinetics by ensuring a high off-rate to favor equilibration in a short time, flexibility to ensure a high on-rate, and a free energy of binding that is a few times thermal energy. This scheme eliminates the most labor-intensive process of designing a universal core module^72^ and therefore makes the platform accessible to researchers with minimal training in DNA origami. Additionally, the design allows for easy functionalization through the attachment of nanoparticles and/or biomolecules,^73–77^ via extending oligos from the core on the upper and bottom portions (Figure S2, Supporting Information sequence).

We quantified the flexibility of dimerized subunit–subunit pairs using cryo-EM, directly visualizing the probability distribution of bending angles between adjacent subunits and subsequently extracting the bending elastic modulus.^29,58^ This angle probability distribution provides valuable insights into how bond flexibility influences the assembly of higher-order structures. Our findings suggest that incorporating an optimal degree of flexibility enhances assembly efficiency and increases error tolerance in both design and fabrication while still delivering high yields and specificity for targeted structures. If excessive flexibility results in unintended assemblies, specificity can be regained by employing additional distinct subunit species (Figure S14).^31–34^ Our related work^58^ on using cryo-EM to assess bond flexibility provides an in-depth technical discussion on the method and links mechanical properties at the subunit scale to accurate predictions of assembly outcomes. Future studies of the cryo-EM method should explore how cryo-EM sample preparation conditions affect structural properties and validate whether oxDNA simulations accurately represent the temperature dependence observed in ssDNA origami structures.

Overall, we demonstrated the versatility of the modular concept by engineering several self-assembled structures with different Gaussian curvatures, suggesting that the method is general and applicable to a wide variety of self-assembled structures imagined by the readers. Interested readers are referred to our follow-up work, which builds on the same concept but uses dsDNA overhangs.^78^ Although dsDNA overhangs require a more complex design and greater expertise, their rigidity could provide more precise angle control. While we realized the concept using DNA origami, the modular engineering principle could also be applied more broadly to other self-assembling systems,^79–81^ opening up new possibilities in nanoscale engineering and materials science.

## Methods

### Design of the DNA Origami Subunits

The subunit core module, made from DNA origami, was designed using caDNAno v2.4 ^82^ (Figure S2), based on multilayer concepts^28,83^ and folded through a one-pot reaction procedure.^84^ The design uses 204 synthetic short ssDNA oligos (staples) to hybridize with and to “fold” one long single-stranded circular DNA (scaffold) into the target structure. The sequences for all core oligos can be found in the Supporting Information Sequence file (please also consult the Nanobase structure #247 ^49^). Twenty-four out of the total 204 ssDNA oligo strands (Figure S2 pink- and cyan-labeled strands; exemplary sequences in Figure S1A) were extended, adding poly-T nucleotides segment (the angle module) and “sticky end” segment (the bond module) to encode geometry and binding information for assemblies, respectively (Figure 1C right). These 24 strands (4 top and 4 bottom strands on each of the three core faces, Figure 1D) are the only part that needs to be modified to make distinct subunit species.

To ensure the binding between subunits has the correct orientation, sequences of the 4 top strands were designed to be different from those of the 4 bottom strands. Additionally, the arrangement of the strands along a row establishes a chirality to each triangle, e.g., a particular row might have the binding sequence a, b, b*, a* with the same sequence repeated on each face (Figures S1B and S3). The symmetry-breaking and chirality prevent flipping of subunits, thereby defining the inside and outside surfaces when assembled into closed structures. To avoid having the faces bind with an offset, all 4 top strands (and all 4 bottom strands) were assigned with different sequences so that binding in any configurations different from the designed one will be weak and therefore transitory while the correct configuration will be strong and long lasting.

Selection of the number of binding strands per subunit face and the number of hybridization pairs (sticky end length) per strand was set by several criteria. (1) The total number of sites to extend ssDNA from the core was limited, with 4 per row being near the maximum. (2) One wants to maximize the off-rate of subunit unbinding, which allows monomers that bind in incorrect positions to fall off, rebind, and equilibrate the assembly into the designed structure. Maximizing the off-rate, which is independent of subunit concentration, is done by reducing the binding strength by decreasing the number of bases involved in the hybridization bond module. (3) One can only reduce the binding strength so much as it is necessary for the binding free energy to exceed *k*_B_ *T* for assembly. The binding free energy is a monotonic function of the ratio of the on- to off-rate constant; therefore, if we fix the binding free energy to be several *k*_B_ *T*, then we must compensate by increasing the on-rate as we increase the off-rate. We do so by increasing the monomer concentration as much as practical (typically ranging 5–100 nM). As shown in Figure S8B, higher subunit concentrations accelerate assembly but also increase aggregation, which reduces the overall yield of correctly assembled structures.^19,72^ Given that higher concentrations also increase material costs without ensuring better results, we used a 5 nM subunit concentration. In summary, our design rules are to maximize the number of strands per face, minimize the number of hybridized base pairs per strand and maximize the monomer concentration, while maintaining the binding free energy near several *k*_B_ *T*.

In this study, we put binding strands on the second and the sixth rows of dsDNA comprising the subunit face (Figures 2D and S1, S2) as we employed the binding strands to set the binding angles, and two points determine a straight line. With 2 rows of 4 binding strands per side of a triangular subunit, we found that assembly was optimized with 5–7 binding base pairs per strand (Figure S5B). To minimize cross-talk between nonpaired segments, we employed a custom sequence optimization procedure to maximize specificity. The algorithm was introduced by Seeman^40^ and was described in detail in our prior work.^34^ Once a set of sequences was determined, we then used the nearest-neighbor model^41^ to compute all the binding free energy and chose a subset of sequences that have similar binding strengths.

We also observed a weak correlation between the poly-T segment length in the angle module and the number of hybridized bases in the bond module required for full assembly (Figure S5A). Therefore, after determining the appropriate hybridization length for a given geometry and temperature, we recommend reassessing binding strength if the poly-T length is changed as this can influence both binding strength and assembly behavior. The strategy is straightforward: if assembly yields only monomers or small clusters and not the target structure, binding can be strengthened by lowering the assembly temperature or increasing the number of hybridized bases in the bond module. For reference, we suggest using 5 binding base pairs for *l*_bottom_ = 3 poly-T, 5 base pairs for *l*_top_ < 10 poly-T, and 6 base pairs for *l*_top_ = 10–14 poly-T (Figure S4). We hypothesize that longer angle modules decrease the binding on-rate due to the higher entropic cost of moving the poly-T region away from the bond module bases.

### Folding and Purification of the DNA Origami Subunits

The folding mixtures contained 50 nM p8064 scaffold (Tilibit Nanosystems), staple oligonucleotides (Integrated DNA Technologies) of 200 nM each, and a folding buffer. The buffer contains 5 mM Tris base, 1 mM ethylenediaminetetraacetic acid (EDTA), 5 mM sodium chloride (NaCl), and 15 mM magnesium chloride (MgCl_2_) (Sigma-Aldrich). The folding mixtures were then subjected to a thermal annealing ramp (80 °C for 2 min, 65 °C for 15 min, then cooling with a 1 °C/h rate from 58 to 50 °C) in a thermal cycling device (Bio-Rad Laboratories).

All folded subunits were then gel purified (to remove excess oligonucleotide strands and misfolded aggregates) and concentrated (using ultrafiltration, 100 kDa molecular-weight cutoff Amicon Ultra Centrifugal Filter Unit) before being used for assembly experiments. An exemplary purification agarose gel is shown in Figure S1C, wherein the monomer “band” contains the target species. A NanoDrop microvolume spectrophotometer (Thermo Fisher Scientific) was used to check the subunit concentrations. Both procedures were performed following details previously described.^72^

### Assembly of Subunits into Desired Structures

All self-assembly experiments were conducted with a total subunit concentration of 5 nM. The assembly solutions contained 5 mM Tris base, 1 mM EDTA, 5 mM NaCl, and 20 mM MgCl_2_. The samples were then incubated in a thermal cycling device at 40 °C for 1 h and quenched at roughly 6 °C/min to the target assembly temperature for a certain reaction time. Optimal assembly conditions vary from structure to structure.

The characteristic assembly time for a given structure can be determined through a time-course kinetic study, as demonstrated for the *T* = 4 shell in Figure S8. Typically, assembly is rapid initially, then slows, and eventually plateaus. Improvements in yield and efficiency can be achieved by optimizing kinetics, such as fine-tuning subunit binding strengths along different symmetry directions.^20^

Here, we provide general strategies to efficiently pick optimal parameters when exploring new designs for desired target structures. (1) Assembly solution and temperature: assembly depends strongly on temperature and relatively weakly on the solution magnesium concentration. We suggest beginning with 20 ± 2.5 mM MgCl_2_ and screening assembly temperature within the range of 25–40 °C. As articulated in the main text, we aim for the highest off-rate possible. We find that the optimal assembly typically occurs 1–2 °C below the structure melting temperature, at which binding strengths between subunits are just strong enough to trigger the formation of high-order structures. This can be easily evaluated by negative stain EM (Figure S5C). Note, both lower assembly temperature and higher MgCl_2_ concentration give stronger subunit–subunit binding. (2) Bond modules: based on systematic exploration (Figure S5), segments of 5–6 base pairs were found to be optimal for the chosen temperature range, and 7 base pairs could be used when a stronger binding is required, for example, to promote hierarchical assembly (Figure S4). Users may wish to adjust segment lengths if they plan to use different assembly temperatures—shorter segments for lower temperatures and longer segments for higher temperatures. (3) Angle modules: to determine a proper angle module, we suggest fixing *l*_bottom_ = 3 poly-T and varying *l*_top_ when targeting a positive binding angle, and fixing *l*_top_ = 3 poly-T and varying *l*_bottom_ when targeting a negative binding angle (Figure S5A). To reduce the effort, first evaluate vertex subassembly when varying *l*_top_ or *l*_bottom_, before checking the assembly of whole structures. For example, a 6-fold (hexamer), 5-fold (pentamer), and 4-fold (tetramer) vertex is expected for binding angles of 0°, 41.8°, and 70.7°, respectively. A binding angle between these numbers would yield a mixture of neighboring close structures.

### Assembly: 2D Sheets with Continuous Tiling Pattern

To construct a 2D sheet with continuous tiling without orientational order, we designed one subunit species with an isotropic interaction matrix. For individual subunits, identical angle modules and bond modules are applied to all three triangular faces S1, S2, and S3 of the core module (Figure 2A). The angle modules employ poly-T segment *l*_top_ = *l*_bottom_ = 3 poly-T (Figures S1A and S4). The binding modules employ 5 bps sticky end segment with “sA” sequence detailed in Figure S3. The assembly temperature is 28 °C with an assembly time of 36 h.

### Assembly: 2D Sheets with Periodic Tetramer Tiling

Two subunit species were designed for this case, color-coded as “yellow” and “green” in Figure 2B, and were mixed with a 3:1 number ratio. The angle modules of both species, for all S1, S2, and S3, employ *l*_top_ = *l*_bottom_ = 3 poly-T (Figures S1A and S4). The “yellow” species binding modules employ 5 bps “A” sequence for S1, “A*” sequence for S2, and “B” sequence for S3 (Figure S3). The “green” species binding modules employ 5 bps “B*” sequence for all S1, S2, and S3. The assembly temperature is 28 °C with an assembly time of 36 h. Note, precise control over system temperature and subunit concentration is suggested to play a dominant role in achieving high-quality, high-yield tiling, while the secondary factor of slight deviations from the optimal subunit ratio is not anticipated to have a major impact on the assembly outcome under the conditions tested.

### Assembly: 3D Small Spherical Shells (*T* = 1 Capsids)

One subunit species was designed, with identical angle module and bond module for all its S1, S2, and S3 (Figure 2C). The angle modules employ *l*_top_ = 14 poly-T > *l*_bottom_ = 3 poly-T (Figures S1A and S4). The angle modules were chosen based on the design that produced the highest capsid yield (Figure S5A), while ensuring that the binding angle fluctuation range (Figure 3A) does not overlap with the next possible closed polyhedral structure (Figure S13A), thereby maintaining target specificity. The binding modules employ “sA” sequence (top 6 bps, bottom 5 bps) (Figure S3). Note, the binding matrix used here is the same as the one for “2D sheets with continuous tiling pattern”. The assembly temperature is 25 °C with an assembly time of 24 h.

### Assembly: 3D Large Spherical Shells (*T* = 4 Capsids)

Two subunit species were designed, color-coded as “yellow” and “green” in Figure 2D, and were mixed with a 3:1 number ratio. For the “yellow” species, angle modules employ *l*_top_ = 14 poly-T > *l*_bottom_ = 3 poly-T for S1 and S2, and *l*_top_ = *l*_bottom_ = 3 poly-T for S3 (Figures S1A and S4); the bond modules employ “A” sequence (top 6 bps, bottom 5 bps) for S1, “A*” sequence (top 6 bps, bottom 5 bps) for S2, and 7 bps “B” sequence for S3 (Figure S3). For the “green” species, angle modules employ *l*_top_ = *l*_bottom_ = 3 poly-T for all S1, S2, and S3 (Figure S1A and S4); the bond modules employ 7 bps “B*” sequence for all S1, S2, and S3 (Figure S3). Here, the yellow-green (B–B*) bond was designed to be stronger than the yellow–yellow (A–A*) bond to induce hierarchical assembly,^20^ biasing formation of tetramer subassemblies. Note, the binding matrix used here is the same as the one for “2D sheets with periodic tetramer tiling”. The assembly temperature is 30 °C with an assembly time of 96 h.

### Assembly: Short Tubes

To construct 3-layer short tubes, we designed three subunit species, color-coded as “yellow”, “green”, and “purple” in Figure 2E, and were mixed with a 1:1:1 number ratio. The angle modules of all three species employ *l*_top_ = 14 poly-T > *l*_bottom_ = 3 poly-T for S1 and S2, and *l*_top_ = 3 poly-T < *l*_bottom_ = 8 poly-T for S3, except the “purple” species whose S3 has no angle modules attached (Figures S1A and S4). The “yellow” species bond modules employ the “sA” sequence (top 6 bps, bottom 5 bps) for S1, the “sB” sequence (top 6 bps, bottom 5 bps) for S2, and the 5 bps “A” sequence for S3. The “green” species bond modules employ “B” sequence (top 6 bps, bottom 5 bps) for S1, “C” sequence (top 6 bps, bottom 5 bps) for S2, and 5 bps “A*” sequence for S3. The “purple” species bond modules employ “B*” sequence (top 6 bps, bottom 5 bps) for S1, “C*” sequence (top 6 bps, bottom 5 bps) for S2, and no bond modules for S3 (Figure S3). The assembly temperature is 30 °C with an assembly time of 96 h.

### Conjugate Gold Nanoparticles to DNA Origami Subunit

The gold nanoparticles (AuNPs, Ted Pella), 10 nm in diameter, were first functionalized with thiol-modified ssDNA (5′-HS-C_6_H_12_-TTTTTAACCATTCTCTTCCT-3′, Integrated DNA Technologies) following scheme described in ref 85. The targeting subunits were also labeled with ssDNA handles with complementary sequence (5′-AGGAAGAGAATGGTT-3′) on their interior edges, following descriptions detailed in ref 49. We first assembled subunits into the desired high-order structures using optimal assembly conditions. The sample solution was then mixed with the AuNP suspension, with a final particle concentration five times larger than the concentration of targeting subunits. The mixture was incubated for 12 h in the native buffer condition before imaging.

### Negative Stain Electron Microscopy

The samples were first prepared using FCF400-Cu grids (Electron Microscopy Science, glow discharged at −20 mA for 30 s at 0.1 mbar using Quorum Emitech K 100× glow discharger before usage) and 2 wt % uranyl formate solution. The images were taken by using an FEI Morgagni Transmission Electron Microscope, operated at 80 kV, with a Nanosprint5 complementary metal-oxide semiconductor camera (AMT). Images were acquired between ×8000 and ×22,000 magnification.

### Agarose Gel Electrophoresis

The assembly yields were investigated by using agarose gel electrophoresis. We employed 0.5 wt % agarose gels containing 0.5× TBE, 3.75% SYBR-safe DNA gel stain, and 20 mM MgCl_2_. The gel electrophoresis was performed at 80 V bias voltage at 4 °C, for varying time (2–4 h) with buffer exchanged every 40 min. The gels were then scanned using a Typhoon FLA 9500 laser scanner (GE Healthcare) at a 25 μm resolution (Figure S6).

The intensity profile of each gel lane was extracted with the background signal subtracted (as obtained from the profile of an empty lane). The processed intensity profile was then normalized and fitted with a Gaussian to the target structure peak. The ratio of the area underneath the Gaussian and the area underneath the whole intensity profile was then defined as the yield (fraction) of successful assemblies.

Each gel lane contains approximately 10^9^ particles, allowing densitometry profiles to provide robust statistical information about assembly population distributions. Band migration distances were compared to standards and between samples to infer species sizes. By correlating these gel bands (Figure S6) with the most common structures observed by EM (on the order of 10^2^ to 10^3^ particles; Figure S7), we confidently assigned bands to specific assemblies. Since our system generally follows a nucleation-and-growth process, only a few dominant species, typically small clusters and closed structures, are present at equilibrium. We therefore focused on determining whether these prevalent species correspond to the target assembly or to off-target closed structures.

### Cryogenic Electron Microscopy

Higher concentrations of DNA origami subunits were used for cryo-EM grids in comparison to those for assembly experiments. To prepare samples, we typically prepared 1–2 mL of folding mixture, gel purified the mixture, and concentrated the sample by ultrafiltration. EM samples were prepared on glow-discharged C-flat 1.2/1.3 400 mesh grids (Protochip). Subunits with a single active bond were prepared and suspended in a buffer containing 5 mM Tris base, 1 mM EDTA, 5 mM NaCl, and 5 mM MgCl_2_. To ensure that dimers formed before plunging, the subunit solution was mixed 1:1 with 35 mM MgCl_2_, bringing their salt concentration to 20 mM MgCl_2_. The solution then sat at room temperature for 30 min. Plunge-freezing of grids in liquid ethane was performed with an FEI Vitrobot VI with sample volumes of 3 μL, wait time of 60 s, blot time of 9 s, and blot force of 0 at 22 °C and 100% humidity.

Cryo-EM images for DNA origami dimers were acquired with a Tecnai F20 TEM with a field emission gun electron source operated at 200 kV and a Compustage, equipped with a Gatan Oneview CMOS camera. Particle acquisition was performed with SerialEM. The defocus was set between −1.5 and −4 μm for all acquisitions with a pixel size of 3.757 Å.

### Single-Particle Reconstruction and Multibody Refinement

Image processing is performed using RELION-4.^86^ Contrast-transfer-function (CTF) estimation is performed using CTFFIND4.1.^87^ After picking single particles (subunits), we perform a reference-free 2D classification from which the best 2D class averages are selected for processing and estimated by visual inspection. The particles in these 2D class averages are used to calculate an initial 3D model. A single round of 3D classification is used to remove heterogeneous monomers, and the remaining particles are used for 3D autorefinement and postprocessing. The postprocessed maps are deposited in the Electron Microscopy Data Bank.

Fluctuations of subunits were processed using RELION-4’s multi-body refinement.^29^ After getting a postprocessed reconstruction of a dimer using single-particle reconstruction, we create masks around the two triangular cores using the eraser tool in ChimeraX.^88^ These were used in the “3D multi-body” job in RELION 4 to get the set of fluctuations the two bodies in the dimer. Outputs of the multibody refinement are the PCs of the fluctuations of the two bodies and corresponding density maps for the two bodies for different eigenvalues along the eigenvectors of the PCs. By measuring the binding angles of the dimers with respect to each other in the PC density maps, we can relate the PC eigenvalues for a given eigenvector to a binding angle of the dimer. This eigen-space to real-space analysis is shown in Figure S11.

### Modeling of the Dimer Binding Angle Distribution Using oxDNA Simulations

Molecular dynamics (MD) simulations of the dimeric modular designs were carried out using the oxDNA2 package.^54^ The initial configuration files for the caDNAno subunit core module were generated using TacoxDNA tools and an in-house script.^89^ Rigid-body dynamics in oxView were performed to align the core module structure into a conformation that better represents the correct global structure.^90,91^ The overhanging ssDNA strands (i.e., the angle and bond modules) were created protruding from the core module using oxView.^90,91^ The resulting structure for each design was duplicated in oxView to form a dimer with parallel core modules connected through the angle and bond modules, thus creating the initial structure for subsequent MD simulations.

MD simulations were preceded by a minimization and an equilibration stage, during which mutual traps between paired scaffolds and staple bases were applied. The structures underwent 10,000 steps of gradient descent minimization followed by a dynamic relaxation. The initial stages of dynamic relaxation involved substituting the DNA backbone potential with linear springs while maintaining mutual traps, where the maximum applied spring force was gradually increased over 1.52 ns to a force value of 57.09 nN/nm with a small time step of Δ*t* = 0.0303 fs. Subsequently, the backbone spring potential was maintained at 57.09 nN/nm, while the time step was increased from Δ*t* = 0.0303 fs to Δ*t* = 9.09 fs over 321.18 ns, with the mutual traps still in place. Lastly, in the final stage of dynamic relaxation, the spring force acting on the backbone was removed, enforcing the full finitely extensible nonlinear elastic potential, while the mutual traps on the base pairs were maintained for 90.9 ns at Δ*t* = 9.09 fs.

A production stage was carried out on the relaxed configurations, where mutual traps were removed, and the dimers were simulated for 0.909 μs at Δ*t* = 9.09 fs at a monovalent salt concentration of 1 M. Note that this simulation time does not directly correspond to physical time due to the implicit treatment of the solvent and the coarsened resolution of the DNA model, which effectively smooths the energy landscape.^92^ Using a previously derived scaling factor (α ≈ 330) from diffusion data, the simulation time in this study corresponds to approximately 300 μs of physical time.^92^ The John thermostat with a diffusion coefficient and Newtonian step settings of 2.5 and 103 was used to maintain a constant temperature (room temperature). Coordinates were stored for 1000 frames in a trajectory file for subsequent analysis, which were conducted using a combination of oxDNA analysis tools and in-house scripts.^91^

The binding angle between neighboring subunits was computed by using a custom script that processes production-stage trajectory files. This script takes the indices of nucleotides on the face of the core module where the overhangs are extended (helix 0 and 4; Figure S2). It also takes as input the indices for two nucleotides from one of the core module’s faces, selected to establish a consistent positive direction vector. These nucleotides were chosen to point from helix 4 to helix 0 along the cross-section of the core module. The index selection was carried out in oxView.^89^ To define the directions along the length and width of the core module face, we computed the first two PCs of each face, from which the core modules are connected, were computed. Their corresponding eigenvectors determined the directions along the width and height of each face, respectively. A vector normal to the width and height directions of each face was derived, and its direction was adjusted to define the binding angle consistently. The binding angle was then calculated using the dot product of the normal vectors, with the sign corrected based on the predefined positive vector pointing from helix 4 to helix 0. This calculation was carried out for the 1000 recorded frames in the trajectory file, resulting in the distribution of binding angles for the two tested designs (Figure S10).

## Supplementary Material









## Data Availability

The authors declare that the data supporting the findings of this study are available within the text, including the Methods section and Supporting Information files. The cryo-EM data associated with dimerized subunits in this study are available on the Electron Microscopy Data Bank (EMD-49952 and EMD-49953).

## Notes

Design of the subunit angle module, bond module, and subunit core; bond module lookup table; angle and bond module lookup table; choosing the appropriate angle module, bond module, and temperature for *T* = 1 capsid assembly; characterizing assembly yield and specificity using gel electrophoresis; electron micrographs of assembled structures; assembly kinetics of *T* = 4 capsid shells; reliable target formation despite bond design variation; simulated dimer fluctuations; mechanical property of the subunit-subunit joint characterized by cryo-EM; twisting and stretching modes of the subunit-subunit joint characterized by cryo-EM; smallest-allowed closed structure; and selectively enriching 5-fold or 6-fold structure using subunits with the same flexible angle module (PDF)
Core module oligo sequence (SI sequence) (XLSX)
Dimer bending fluctuation reconstructed from cryo-EM: angle modules *l*_top_ = *l*_bottom_ = 3 poly-T (AVI)
Dimer bending fluctuation reconstructed from cryo-EM: angle modules *l*_top_ = 14 poly-T > *l*_bottom_ = 3 poly-T (AVI)
