# Supplementary material for: Economical and Versatile Subunit Design Principles for Self-Assembled DNA Origami Structures
Source: ACS Nano. 2025 Aug 19;19(34):30889–901. doi: 10.1021/acsnano.5c06681 (PMC12410049; doi:10.1021/acsnano.5c06681)
Supplement: Supplementary file 1 [file nn5c06681_si_001.pdf]

Supporting Information

Economical and versatile subunit design principles for self-assembled DNA origami structures

Wei-Shao Wei*[1,2], Thomas E. Videbæk[1,2], Daichi Hayakawa[1,2], Rupam Saha[1,2], Juanita Pombo[3], Gaurav Arya[3], W. Benjamin Rogers[1,2], Seth Fraden*[1,2]

[1] Martin A. Fisher School of Physics, Brandeis University, Waltham, MA 02453, USA
[2] Materials Research Science and Engineering Center (MRSEC), Brandeis University, Waltham, MA 02453, USA
[3] Thomas Lord Department of Mechanical Engineering and Materials Science, Duke University, Durham, NC 27708, USA

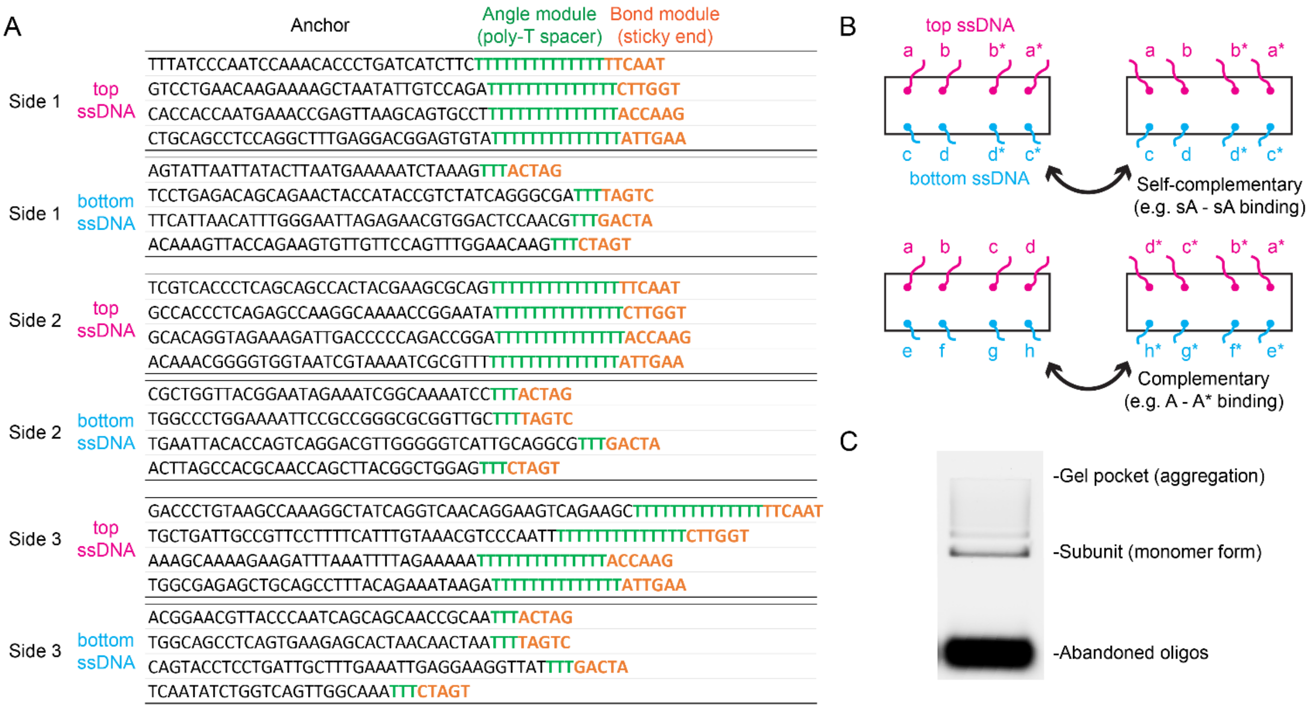

Figure S1. Design of the subunit angle and bond module. (A) 5' to 3' sequences of the total 24 variable ssDNAs. Each strand contains 3 segments – conserved 'anchor' (black), variable angle module (poly-T spacer, green), and variable bond module (sticky end, orange). Here, the angle modules employ ℓ_{top} = 14 poly-T, ℓ_{bottom} = 3 poly-T, the bond modules employ 'sA' sequence (Figure S3, Figure S4) which make $T = 1$ capsids shown in Figure 2C. (B) The organization of 8 ssDNAs on each core face for self-complementary binding (*top*) or for binding between two species (complementary; *bottom*). a-a*, b-b*, etc. indicate complementary ssDNA sequences (Figure S3). (C) An exemplary purification agarose gel, with the band labeled 'subunit (monomer form)' contains the target species.

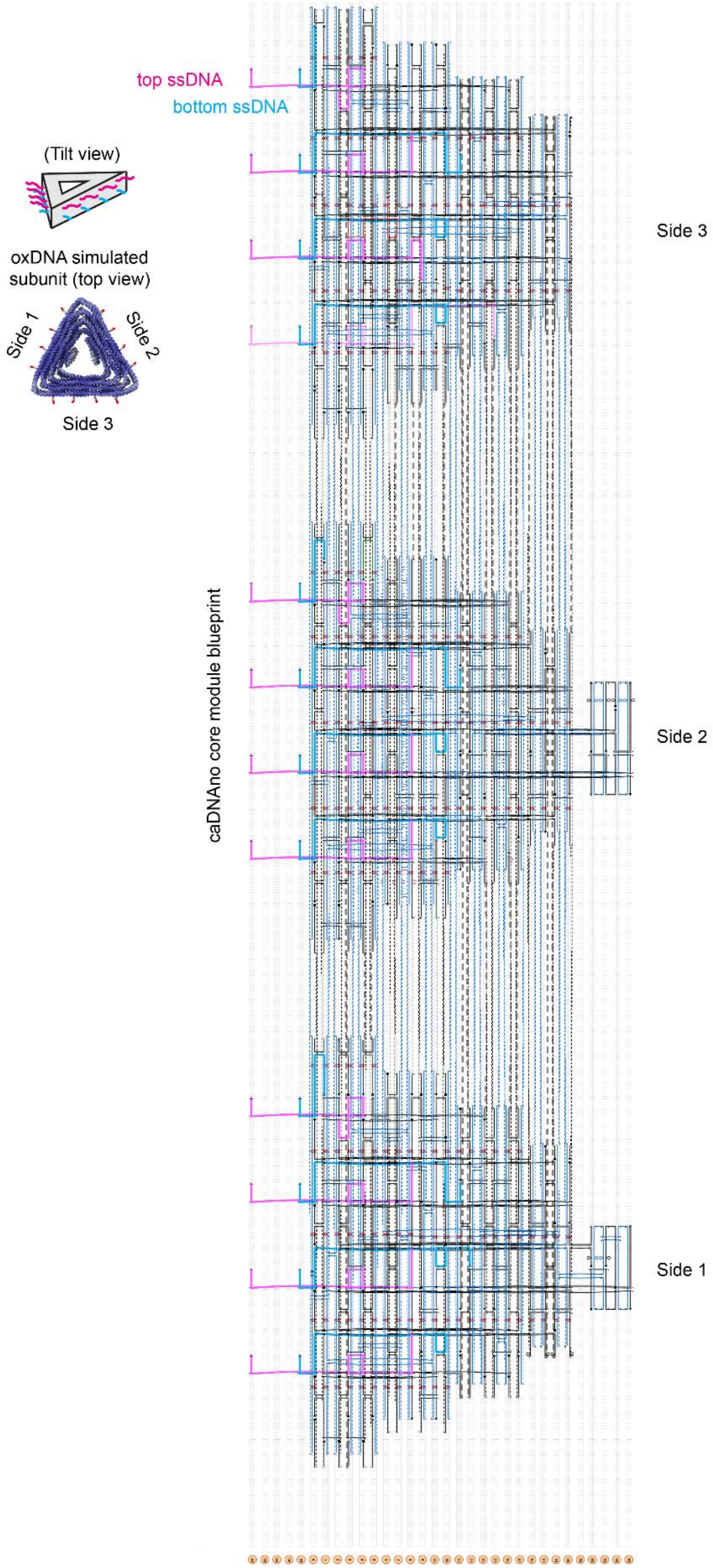

Figure S2. Design of the subunit core. The core module designed using caDNAno, with one circular p8064 scaffold and 204 oligos (see SI Sequence file for the full oligo sequences). On each subunit triangle face/side, 4 top (pink) and 4 bottom (cyan) ssDNA strands comprise the angle and bond module (see Figure S1A-B). Inset: sketch of a tilt-view subunit and an oxDNA-simulated top-view subunit.

		1	2	3	4
**sA**	top	TTCAATT	CTTGGTA	TACCAAG	AATTGAA
	bottom	ACTAGCA	TAGTCTA	TAGACTA	TGCTAGT
**sB**	top	ATAAGT	TCCTTC	GAAGGA	ACTTAT
	bottom	TCAGAC	TACCTT	AAGGTA	GTCTGA
**sC**	top	CCATTC	CTCGAA	TTCGAG	GAATGG
	bottom	AGTTAC	GTCTAG	CTAGAC	GTAACT
**sD**	top	GATCTT	TCCACA	TGTGGA	AAGATC
	bottom	TTAACC	TTGGAT	ATCCAA	GGTTAA
**A**	top	ATGACA	AACCTA	GAGACA	ACTAAC
	bottom	CAATAG	CTAGGA	CACATC	ACCTGA
**A***	top	GTTAGT	TGTCTC	TAGGTT	TGTCAT
	bottom	TCAGGT	GATGTG	TCCTAG	CTATTG
**B**	top	AGATAGTG	TTCCATAC	GATATGTG	AACATTAT
	bottom	GCATCTCA	AGATTCGA	TTCTCACC	TCGTACTC
**B***	top	ATAATGTT	CACATATC	GTATGGAA	CACTATCT
	bottom	GAGTACGA	GGTGAGAA	TCGAATCT	TGAGATGC
**C**	top	GGATAA	GGTATT	GGTAAT	AGAGAT
	bottom	AGTTCC	ATTCTG	ATTCAG	GTAGAT
**C***	top	ATCTCT	ATTACC	AATACC	TTATCC
	bottom	ATCTAC	CTGAAT	CAGAAT	GGAACT
**D**	top	GAGACA	GACAGA	TACAGG	AACCTA
	bottom	CACATC	ACGAAG	TGATTG	CTAGGA
**D***	top	TAGGTT	CCTGTA	TCTGTC	TGTCTC
	bottom	TCCTAG	CAATCA	CTTCGT	GATGTG
**E**	top	GATATG	ATGCAC	TTCCTG	TTCCAT
	bottom	TTCTCA	CTGTGA	TATTCC	AGATTC
**E***	top	ATGGAA	CAGGAA	GTGCAT	CATATC
	bottom	GAATCT	GGAATA	TCACAG	TGAGAA
**F**	top	GGTAAT	ACTGAG	TCATCC	GGTATT
	bottom	ATTCAG	CTTGAG	CGATTA	ATTCTG
**F***	top	AATACC	GGATGA	CTCAGT	ATTACC
	bottom	CAGAAT	TAATCG	CTCAAG	CTGAAT

Figure S3. Bond module lookup table. The 4 top and 4 bottom sticky end sequences (5' to 3') are provided for bond module. Any set of 8 sequences can be applied to any core face. As an example, subunits with all sides equipped with the 'sA' sequences (top 6 bps; bottom 5 bps) are good for T=1 capsid assembly (shown in Figure S1B orange sequence segment). Consult Figure S4 for how to use the sequences listed in the table to build other assembly structures described in this work. The sA, sB, etc. sequence sets can be used when self-complementary bindings are needed between subunits (with the two jointed subunit faces having the same sequence; Figure S1C *top*). The A-A*, B-B*, etc. sequence sets can be used for binding between two different subunit species (with the two jointed subunit faces having different sequences; Figure S1C *bottom*). Here we provide binding sequences with varying lengths that can be used for desired binding strength. Use black, black + red, black + red + blue, or black + red + blue + purple sequences for sticky ends of 5, 6, 7, or 8 bps long (weak to strong binding), respectively.

A

Assembly structure	Subunit	Target binding angle (S1, S2, S3)		Angle module (S1, S2, S3)	Bond module (S1, S2, S3)	
					Sequence	bp number
2D sheet with contenious tiling	single species	0°, 0°, 0°	top	3, 3, 3 poly-T	sA, sA, sA	5, 5, 5 bps
			bottom	3, 3, 3 poly-T	sA, sA, sA	5, 5, 5 bps
2D sheet with tetramer tiling	yellow species	0°, 0°, 0°	top	3, 3, 3 poly-T	A, A*, B	5, 5, 5 bps
			bottom	3, 3, 3 poly-T	A, A*, B	5, 5, 5 bps
	green species	0°, 0°, 0°	top	3, 3, 3 poly-T	B*, B*, B*	5, 5, 5 bps
			bottom	3, 3, 3 poly-T	B*, B*, B*	5, 5, 5 bps
T=1 capsid shell	single species	41.8°, 41.8°, 41.8°	top	14, 14, 14 poly-T	sA, sA, sA	6, 6, 6 bps
			bottom	3, 3, 3 poly-T	sA, sA, sA	5, 5, 5 bps
T=4 capsid shell	yellow species	41.8°, 41.8°, 0°	top	14, 14, 3 poly-T	A, A*, B	6, 6, 7 bps
			bottom	3, 3, 3 poly-T	A, A*, B	5, 5, 7 bps
	green species	0°, 0°, 0°	top	3, 3, 3 poly-T	B*, B*, B*	7, 7, 7 bps
			bottom	3, 3, 3 poly-T	B*, B*, B*	7, 7, 7 bps
Short tube	yellow species	41.8°, 41.8°, -21.6°	top	14, 14, 3 poly-T	sA, sB, A	6, 6, 5 bps
			bottom	3, 3, 8 poly-T	sA, sB, A	5, 5, 5 bps
	green species	41.8°, 41.8°, -21.6°	top	14, 14, 3 poly-T	B, C, A*	6, 6, 5 bps
			bottom	3, 3, 8 poly-T	B, C, A*	5, 5, 5 bps
	purple species	41.8°, 41.8°, N/A	top	14, 14, N/A poly-T	B*, C*, N/A	6, 6, N/A bps
			bottom	3, 3, N/A poly-T	B*, C*, N/A	5, 5, N/A bps

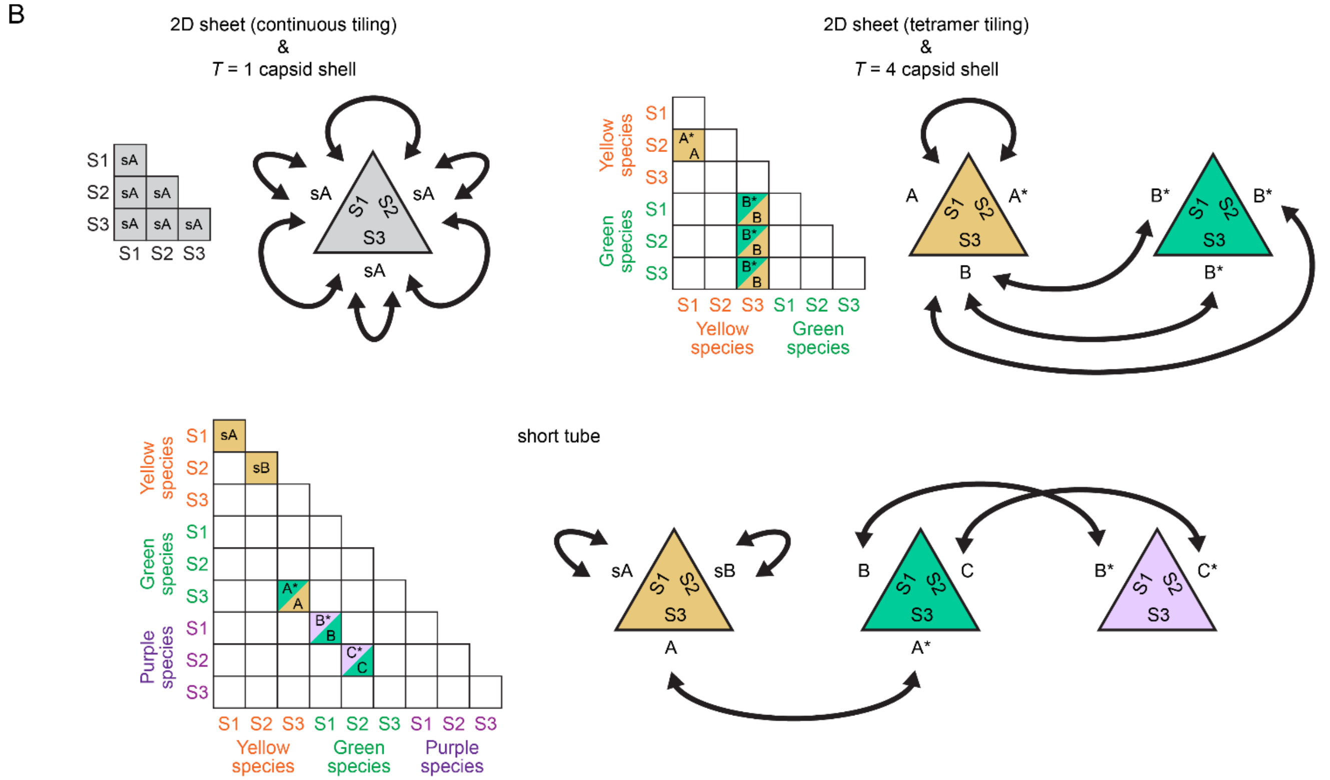

Figure S4. Angle and bond module lookup table, as well as interaction rules, for various assembly structures. (A) Here we provide detailed choices of angle modules (green) and bond modules (orange) to synthesize specific subunits for assembly of 2D sheets, *T*=1 and *T*=4 capsids, and short tubes described in Figure 2. Any chosen angle and bond modules will appear as extension of the conserved 'anchor' segment as detailed in Figure S1A. The sequences of bond modules can be found in Figure S3. See Methods for detailed design strategies. Note, the S3 of purple species of short tube do not have connected neighbors, and thus no angle/bond modules are equipped (denoted as N/A). (B) Illustrations of the interaction matrices are provided for the following assemblies: 2D sheet with continuous tiling and *T* = 1 capsid (*upper left*; Figure 2A, 2C 2*nd* *column*), 2D sheet with tetramer tiling and *T* = 4 capsid (*upper right*; Figure 2B, 2D 2*nd* *column*), and short tube (*bottom*; Figure 2E 2*nd* *column*). Letters indicate distinct bond sequences, as detailed in Figure S3.

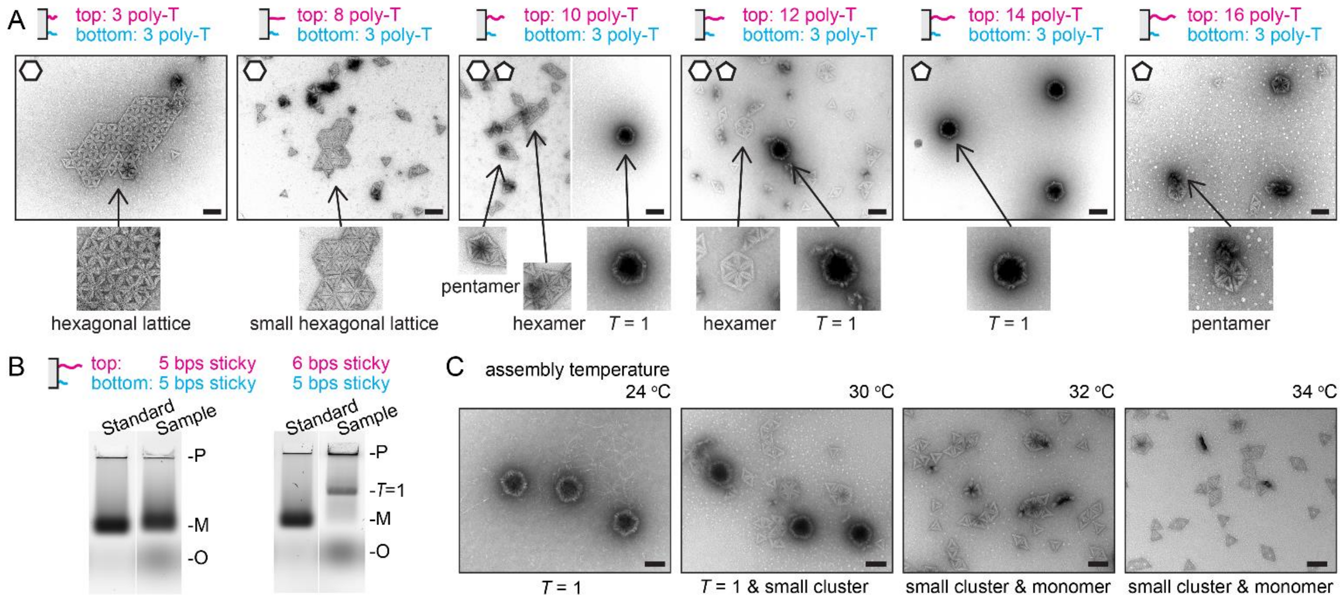

Figure S5. Choosing the appropriate angle module, bond module, and temperature for $T = 1$ capsid assembly. (A) An appropriate angle module could be determined through systematic trials, with a fixed core module, fixed bond module, fixed $\ell_{\text{bottom}} = 3$ poly-T, and only varying ℓ_{top}. With increasing ℓ_{top}, the following sequence of assembly structures was observed – large 6-fold planar sheet (3 poly-T), small 6-fold planar sheet (8 poly-T), mixture of 6-fold structures and 5-fold T=1 capsid shell (10 and 12 poly-T), and highly-specific 5-fold T=1 capsid shell (14 poly-T). In the case of $\ell_{\text{top}} = 16$ poly-T, highly-specific 5-fold structures (e.g. pentamers) were observed but the binding strength was too weak to form a closed T=1 capsid shell. Inset: enlarged structures showing details. We thus choose $\ell_{\text{top}} = 14$ poly-T for the optimal $T = 1$ capsid assembly at an assembly temperature of 25 °C. Scale bar: 100 nm. (B) An appropriate bond module includes well-designed binding base pair sequences and suitable binding base pair numbers (sticky end lengths). The former was discussed in Figure S3, and the latter could be determined through systematic trials, with a fixed core module, fixed angle module ($\ell_{\text{top}} = 14$ poly-T, $\ell_{\text{bottom}} = 3$ poly-T), fixed bond module sequence, fixed assembly temperature (e.g. 25 °C), and only varying sticky end lengths. The binding strength should be 'just enough' for high-order assembly; below which subunits only form small clusters but no larger structures (*left*, using 5 bps for both top and bottom overhanging ssDNA sticky ends), and above which target assembly can form (*right*, using 6 bps and 5 bps for the top and bottom sticky ends, respectively). The agarose gel is used to characterize product contents, with 'P', 'T=1', 'M', and 'O' indicate large aggregation in gel pocket, target T=1 capsid, monomer, and abandoned oligos, respectively. We thus choose the 6 bps / 5 bps combination for the optimal T=1 capsid assembly. (C) Once the subunit structure is determined, the optimal assembly temperature should be picked to be below but near the structure 'melting temperature' (30-32 °C in this case), above which subunits tend to remain as monomers or small clusters. Scale bar: 100 nm.

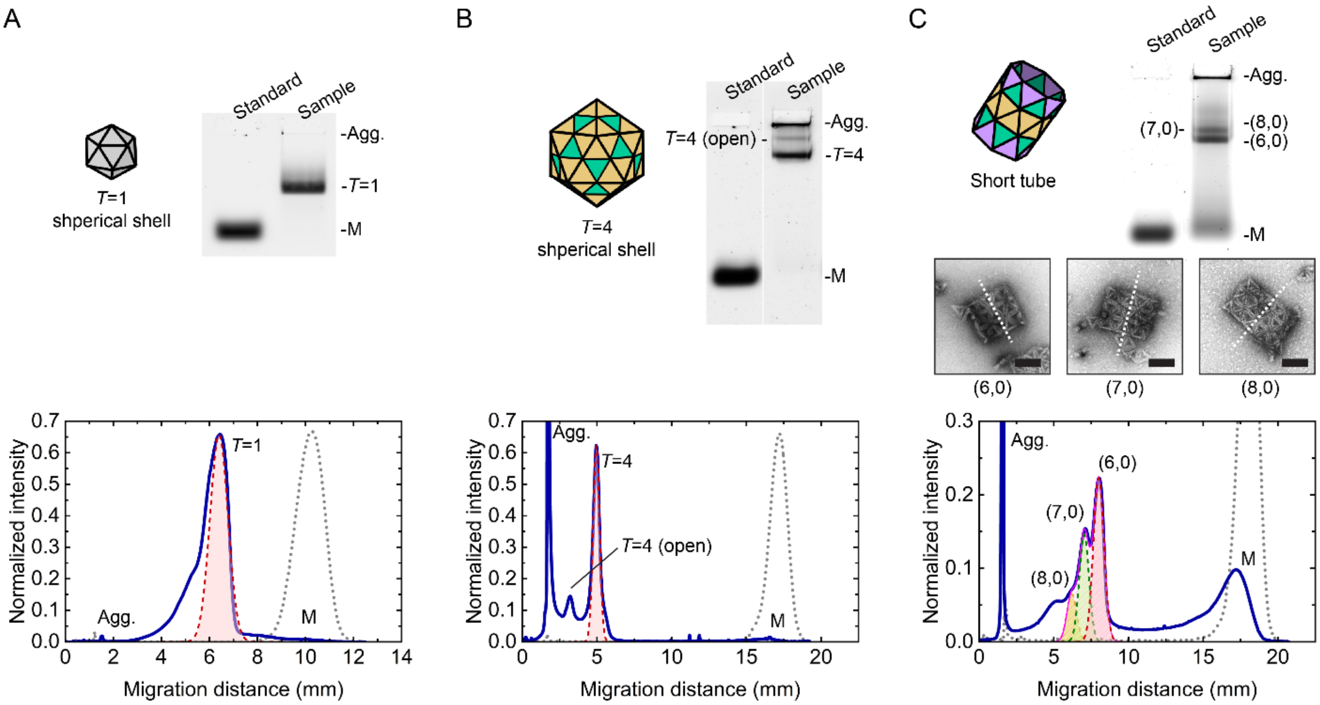

Figure S6. Characterizing assembly yield and specificity using gel electrophoresis. (A-C) Assembly products characterized by gel electrophoresis for three exemplary structures, including (A) $T = 1$ small icosahedral shells (see Figure 2C), (B) $T = 4$ large icosahedral shells (see Figure 2D), and (C) short tubes (see Figure 2E). *Top row*: laser-scanned fluorescent images of agarose gels showing the monomer standard (*left lane*) and the assembly product (*right lane*). The samples are loaded into the top gel pockets and migrate toward the bottom, with smaller species moving faster. The 'Agg.', 'M', '$T = 1$', '$T = 4$', '$T = 4$ (open)', '(6, 0)', '(7, 0)', '(8, 0)' labels indicate the position of aggregation (large structures remaining in the gel pocket), peak of monomer population, $T = 1$ capsid population, $T = 4$ capsid population, opened/cracked $T = 4$ capsid population (see Figure S7C), and three peaks of short tube populations with varying diameter. The (6, 0), (7, 0), and (8, 0) tubes contain 12,14, and 16 subunits alone its ring direction, respectively, and are shown under EM (the white dashed line indicates the axis of symmetry; scale bar: 100 nm). *Bottom row*: the normalized intensity profiles obtained from gels show the spatial distribution and relative species population in each sample. Both the sample (blue solid curves) and the monomer standard (grey dotted curves) are plotted. The $T = 1$ capsid (A), $T = 4$ capsid (B), and (6, 0), (7, 0), (8, 0) tubes (C) population peaks are fitted with a Gaussian (dashed curve with shaded area). The complete target yield is defined as the ratio of the area underneath the Gaussian and the area underneath the blue solid curve.

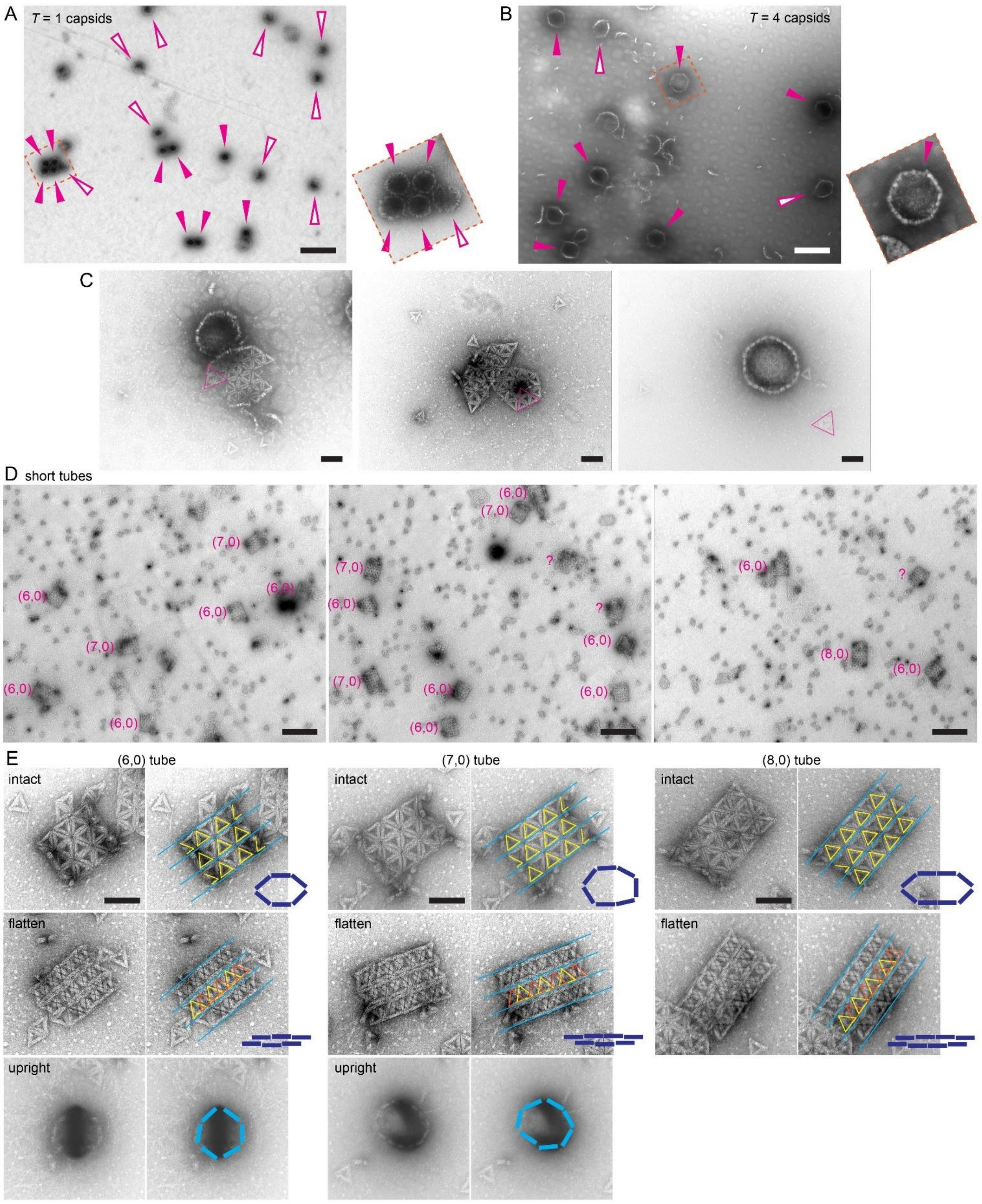

Figure S7. Electron micrographs of assembled structures. Negative stain EM images show (A) $T = 1$ capsids, (B) $T = 4$ capsids, and (D) tubes. Pink solid arrows mark complete capsids; pink hollow arrows indicate cracked or open capsids. Tubes are labeled by lattice number (m, n). Monomers, small clusters, and incomplete structures are also visible. Scale bar: 400 nm. (C) Examples of opened/cracked (*left*), incomplete (*middle*), and closed (*right*) $T = 4$ capsids, each showing tetramer subassemblies. Scale bar: 100 nm. (E) Short tubes with different lattice numbers are shown in intact (*top*), flattened (*middle*), and upright (*bottom*) forms, with sketch overlays to highlight lattice structures. Scale bar: 100 nm.

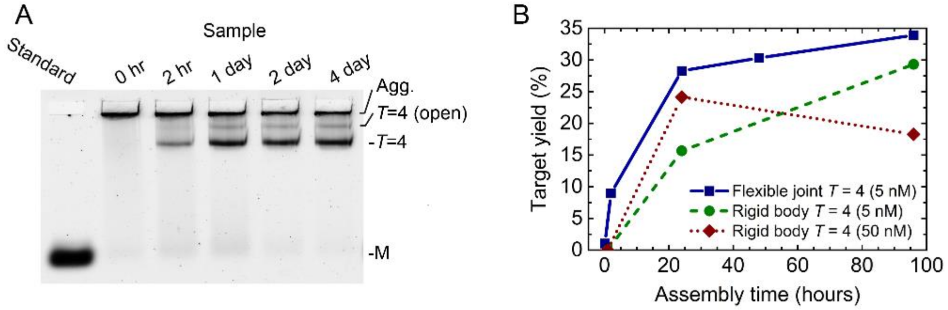

Figure S8. Assembly kinetics of $T = 4$ capsid shells. (A) Laser-scanned fluorescent image of an agarose gel showing a time-course study of $T = 4$ shell assembly. The labels 'Agg.', '$T = 4$ (open)', '$T = 4$', and 'M' indicate the positions of aggregates (large structures remaining in the gel pocket), opened/cracked $T = 4$ capsids (see Figure S7C), target $T = 4$ capsids, and monomers, respectively. The absence of monomer bands in the early time points (e.g. 0 hr, 2 hr) is an artifact of performing gel electrophoresis at room temperature. The lower gel running temperature, compared to the assembly temperature, enhances subunit-subunit interactions, causing monomers and small clusters to rapidly aggregate and remain in the pocket as 'Agg'. However, it isn't possible for closed capsids to grow larger and thus the room temperature gels accurately report the yield of the closed capsid as a function of time. (B) The yield of target $T = 4$ capsids as a function of assembly time, extracted from the gel in (A). The solid blue curve shows rapid assembly within the first 24 hours, followed by a slower reaction that plateaus around 96 hours. For comparison, $T = 4$ capsids assembled from subunits with a fixed-angle (rigid body) design from our prior studies[19,72] (at the same subunit concentration; green dashed curve) assemble more slowly and reach a lower final yield. Increasing the subunit concentration tenfold for the rigid body design (red dotted curve) accelerates early assembly but promotes aggregation, resulting in a lower long-term yield, likely due to nonspecific interactions between subunits.

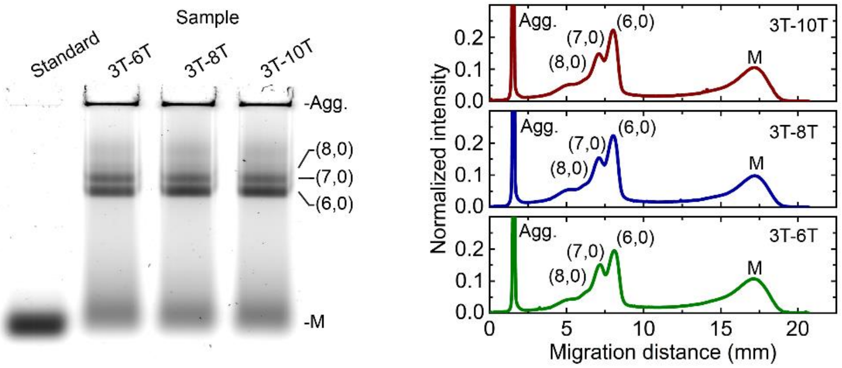

Figure S9. Reliable target formation despite bond design variation. To assess error tolerance, we systematically varied the poly-T segment length in the angle module for short tube assembly (see Figure 2E, Figure S6C). Specifically, we tested $\ell_{top} = 3$ poly-T with $\ell_{bottom} = 6$, 8, or 10 poly-T, and analyzed the resulting assemblies by gel electrophoresis (*left*). Labels 'Agg.', 'M', '(6, 0)', '(7, 0)', and '(8, 0)' indicate aggregates, monomers, and tube populations of different diameters, respectively. The (6, 0), (7, 0), and (8, 0) tubes contain 12, 14, and 16 subunits per ring (see Figure S7E). Results show consistent formation of target structures across these variations, with only minor yield fluctuations (*right*; normalized intensity profiles obtained from the gel). The $\ell_{top} = 3$ poly-T, $\ell_{bottom} = 8$ poly-T design is used throughout this work.

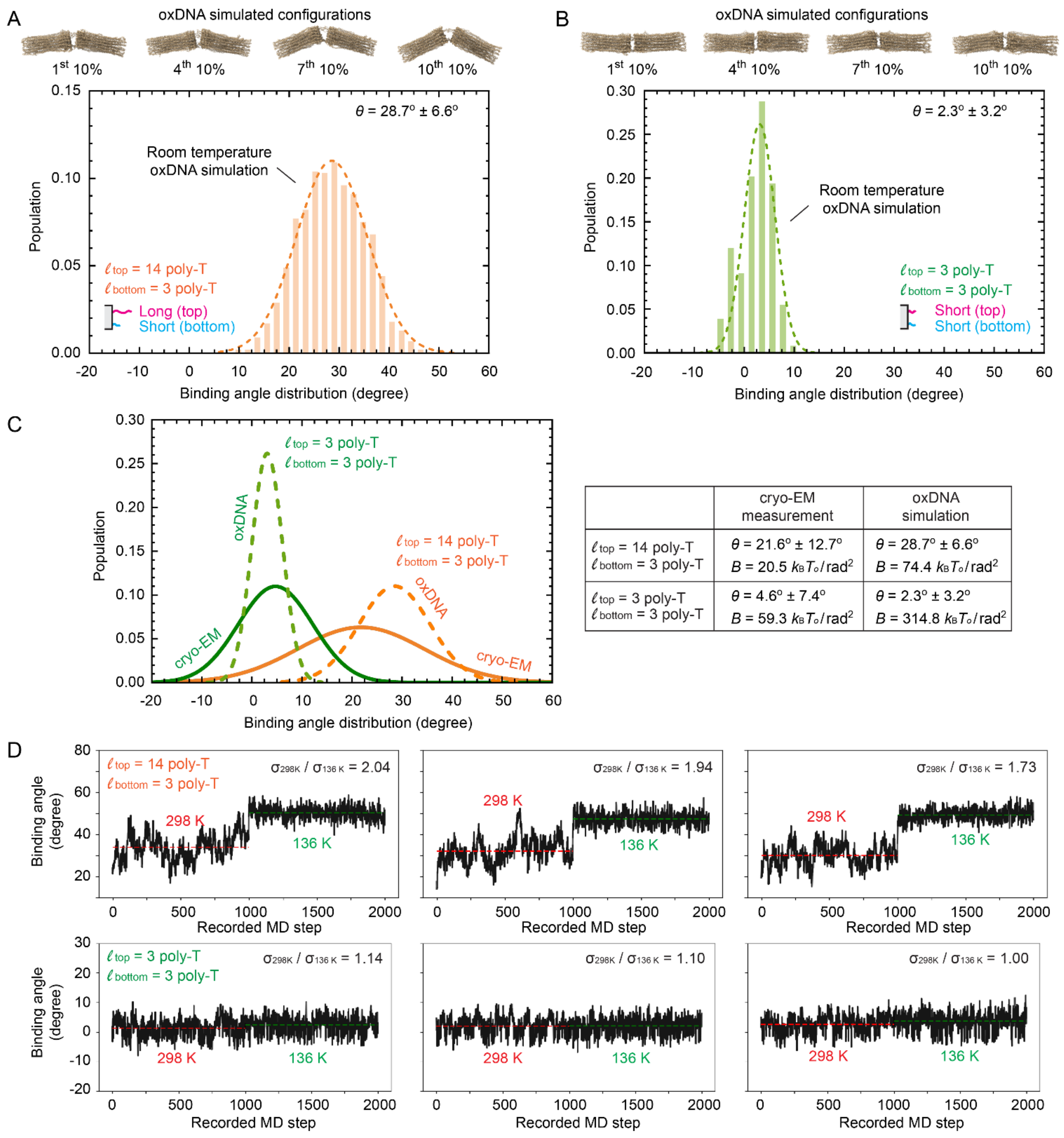

	cryo-EM measurement	oxDNA simulation
ℓ_{top} = 14 poly-T<br>ℓ_{bottom} = 3 poly-T	θ = 21.6° ± 12.7°<br>B = 20.5 k_BT_o/rad^2	θ = 28.7° ± 6.6°<br>B = 74.4 k_BT_o/rad^2
ℓ_{top} = 3 poly-T<br>ℓ_{bottom} = 3 poly-T	θ = 4.6° ± 7.4°<br>B = 59.3 k_BT_o/rad^2	θ = 2.3° ± 3.2°<br>B = 314.8 k_BT_o/rad^2

Figure S10. Simulated dimer fluctuations. The dimerized subunit pair with angle modules (A) ℓ_{top} = 14 poly-T > ℓ_{bottom} = 3 poly-T and (B) ℓ_{top} = ℓ_{bottom} = 3 poly-T are computationally simulated by oxDNA at room temperature. The former design has a broader binding angle distribution (larger standard deviation) than the latter one, demonstrating similar trends as observed in the cryo-EM experiments (Figure 3). (C) Note that the oxDNA distributions are narrower (dashed curves) than the cryo-EM experiments (solid curves, rescaled to room temperature; Figure 3) by roughly a factor of two. Additionally, mean of the oxDNA and cryo-EM distributions are different (*left*). The bending elastic modulus derived from oxDNA simulations are thus greater than the cryo-EM measurements (*right*). (D) The temperature effect on angular distribution, measured by standard deviation σ, was evaluated using oxDNA simulations for both bond designs. *Top*: three parallel simulations with ℓ_{top} = 14 poly-T > ℓ_{bottom} = 3 poly-T yield σ_{298K} / σ_{136K} = 1.90. *Bottom*: three parallel simulations with ℓ_{top} = ℓ_{bottom} = 3 poly-T yield σ_{298K} / σ_{136K} = 1.08. For reference, a pure entropic spring would give a ratio of 1, while a temperature-independent elastic constant would give 1.48. These results indicate that the bond's elastic properties are more complex than either model predicts.

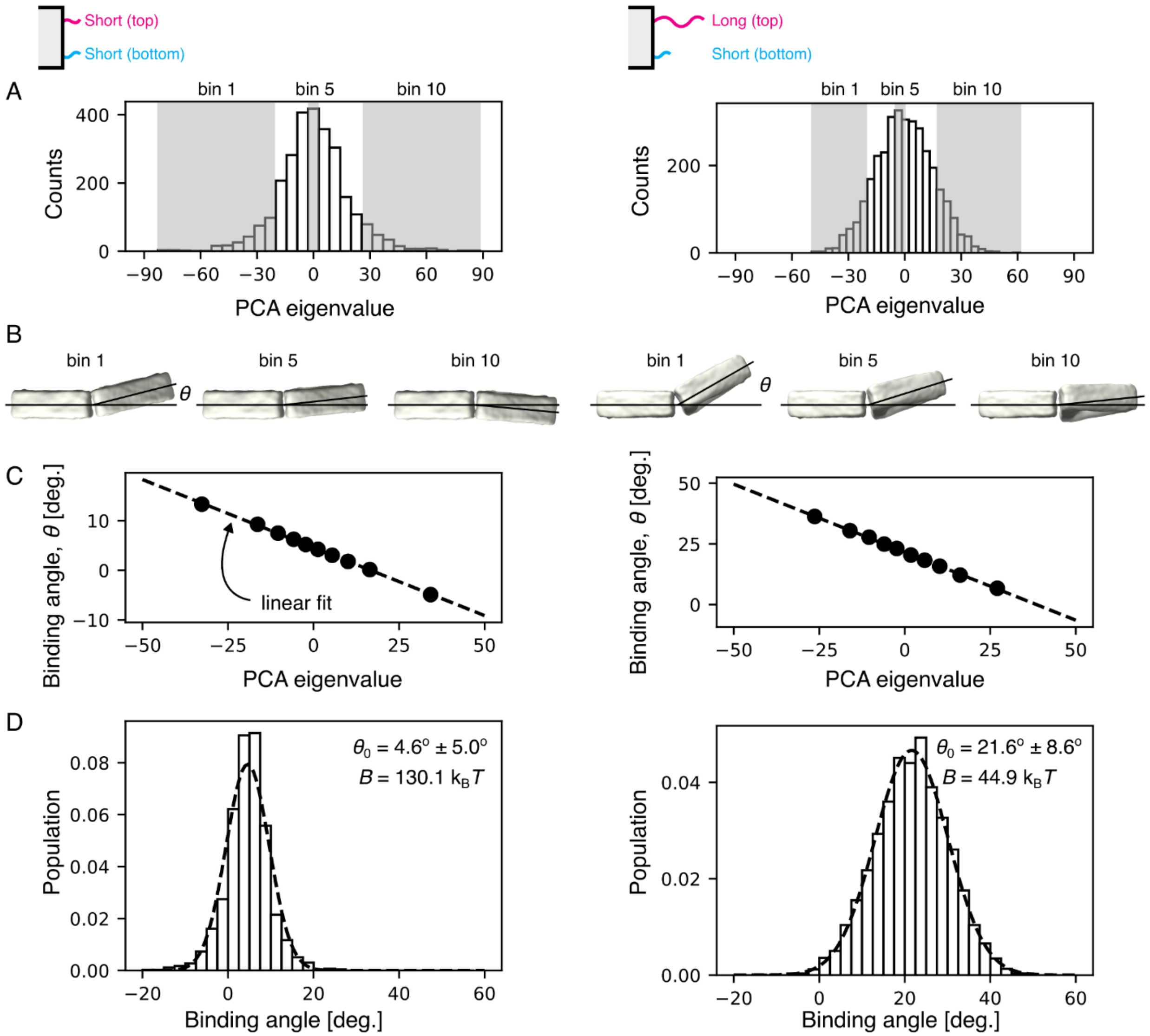

Figure S11. Mechanical property of the subunit-subunit joint characterized by cryo-EM. (A) Histogram of eigenvalues corresponding to the multi-body PCA mode that is closest to a bending mode. The distribution in (A) is divided into ten equi-populated bins and the dimer configuration for the average eigenvalues of the PCA mode is output as a density map, shown in (B) for bins 1, 5, and 10. From these dimer configurations we measure the binding angle along the centerline of the two triangles. (C) Plotting the measured angles and the average eigenvalues for each bin shows that they are linearly related. Fitting these points (*dashed line*) allows us to convert the PCA eigenvalues into opening angles for the dimers. (D) Using the linear relationship, we construct distributions for the binding angle of the dimers. We find that these distributions appear normal, so we fit them to a Gaussians (*dashed curve*). This gives us the average binding angle for the dimer. By assuming that these distributions arise from a Boltzmann distribution of the elastic bending energy, we can estimate a bending elastic modulus as $B=1/\sigma^2$, where σ is the standard deviation of the angle distribution. *Left column*: data for subunits jointed with angle modules $\ell_{\text{top}} = \ell_{\text{bottom}} =$ 3 poly-T. *Right column*: data for subunits jointed with angle modules $\ell_{\text{top}} = 14$ poly-T, $\ell_{\text{bottom}} = 3$ poly-T.

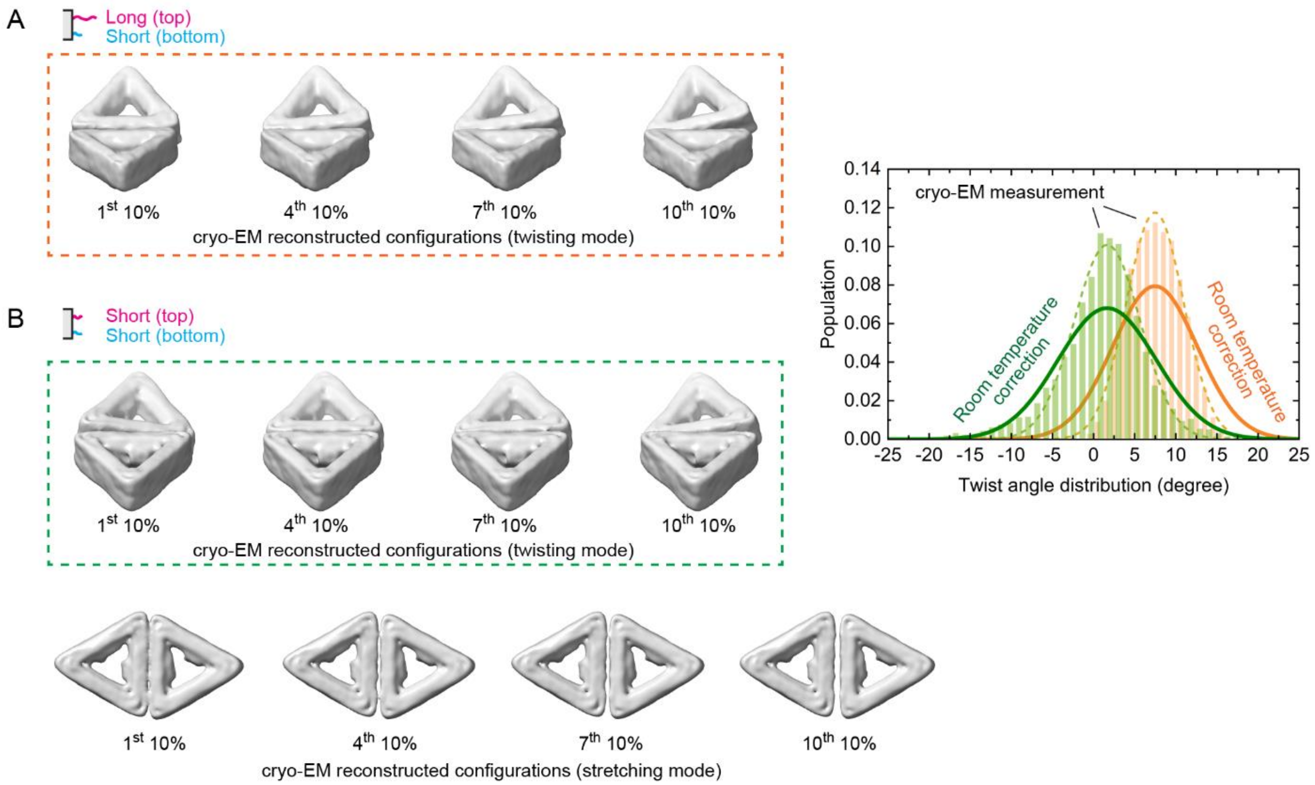

Figure S12. Twisting and stretching modes of the subunit-subunit joint characterized by cryo-EM. (A) The twist mode in dimers with angle modules ℓ_{top} = 14 poly-T > ℓ_{bottom} = 3 poly-T. The four cryo-EM reconstructions represent average configurations of the most clockwise twisted 10% ensemble (negative twist angle), the most counterclockwise twisted 10% ensemble (positive twist angle), and another two states in between. The twist angle distribution is extracted from cryo-EM observation (orange bar graph), assumed to be equilibrated at 136 K, fitted by a Gaussian (dashed orange curve), and rescaled to the room temperature ensemble at 298 K (solid orange curve), giving an estimated room temperature twist elastic modulus of 124.1 k_BT/rad^2. (B) The dimer joined by angle modules $\ell_{top} = \ell_{bottom}$ = 3 poly-T has a twisting mode with an estimated room temperature twist elastic modulus of 92.4 k_BT/rad^2 (green curves and top cryo-EM reconstructions) and stretching mode (bottom cryo-EM reconstructions).

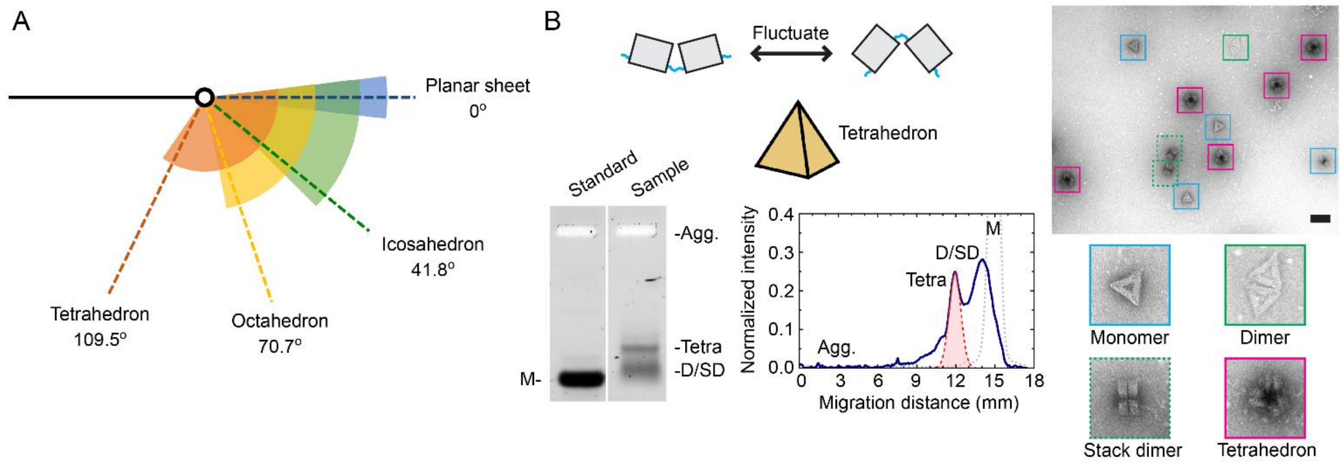

Figure S13. Smallest-allowed closed structure is favorable. (A) Schematic illustrating the idea that if there is more than one possible polymorph within the fluctuation range, the closed structure with the smallest number of subunits is kinetically favorable. The blue, green, yellow, and orange shaded circular sector indicates the range of binding angle fluctuation that would lead to the corresponding closed structure of planar sheet (case in Figure 2A, Figure 3B), icosahedron (case in Figure 2C, Figure 3A), octahedron, and tetrahedron (case in (B)), respectively. (B) A very flexible subunit-subunit joint (orange shaded circular sector in (A)) can be realized by only employing the bottom-row ssDNA strands as the angle/bond modules (*top left*). In this extreme case, subunits favor self-assembling into small tetrahedron (the smallest-allowed closed structure) and dimer, as shown in the EM image (*right*; scale bar: 100 nm) and evaluated by the gel electrophoresis (*bottom left*). 'Agg.', 'Tetra', 'D/SD', and 'M' labels indicate the position of aggregates excluded from entering the gel, peak of the tetrahedron population, peak of the dimer and stack dimer (two monomers stack face-to-face), and monomer population, respectively. The intensity profile shows the spatial distribution and relative species population of the assembly sample (blue solid curve) and the monomer standard (grey dotted curve). The tetrahedron population peak is fitted with a Gaussian (red dashed curves), giving a target yield of 28%. Note that no larger closed structures than tetrahedron were observed, even though the joins are super flexible.

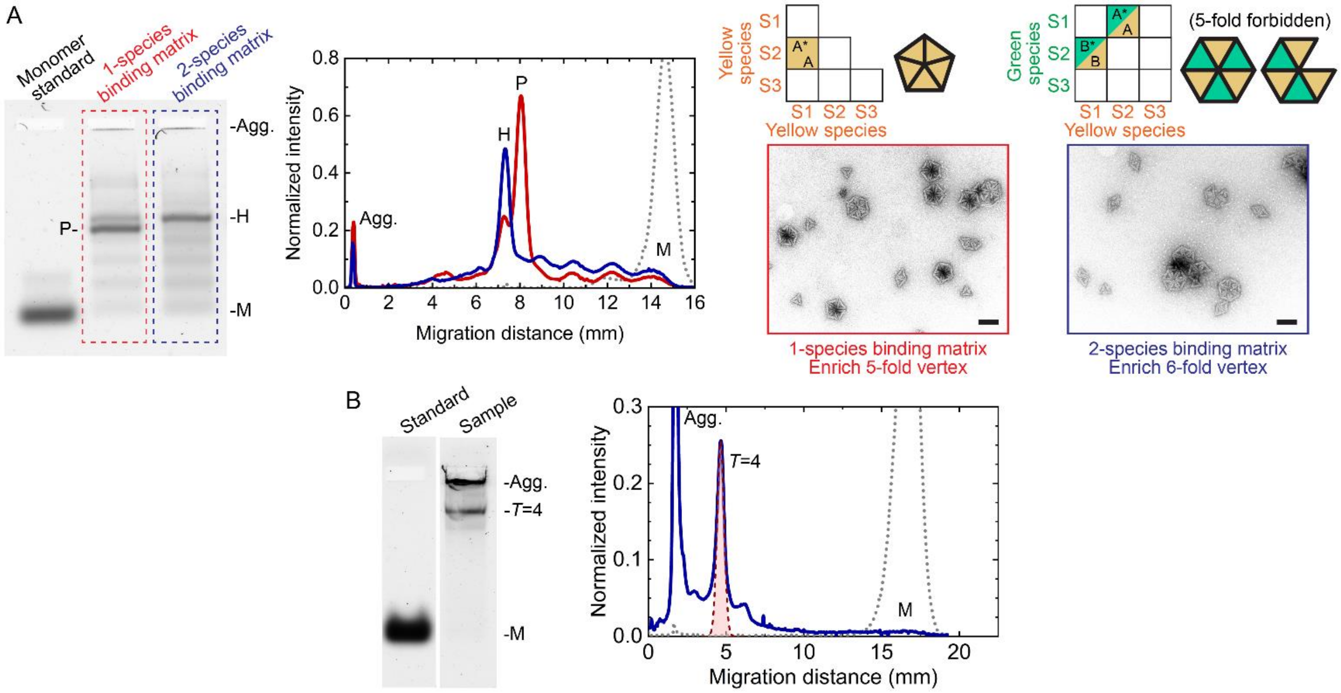

Figure S14. Selectively enriching 5-fold or 6-fold structure using subunits with the same flexible angle module. (A) As shown in Figure 4A, the angle module ℓ_{top} = 14 poly-T, ℓ_{bottom} = 3 poly-T can be programmed to preferentially form different structural binding angles by increasing the number of distinct subunits from one to two. The 1-species binding matrix case (red color-coded) favors forming the 41.8° binding angle that makes a 5-fold vertex. The 2-species binding matrix case (blue color-coded) favors forming the 0° binding angle and 6-fold vertex. The experimental observations are demonstrated in the laser-scanned fluorescent images of agarose gel (*left*; 'Agg.', 'H', 'P', 'M' labels indicate the position of gel pocket/aggregation, peak of hexamer, pentamer, and monomer population, respectively), intensity profiles obtained from the gel (*middle*), and EM images (*right*; scale bar: 100 nm). (B) $T = 4$ capsids assembled from subunits with identical flexible angle modules (ℓ_{top} = 14 poly-T, ℓ_{bottom} = 3 poly-T) on all three bonds. *Left*: laser-scanned fluorescent images of agarose gel showing the monomer standard (*left lane*) and the sample (*right lane*). The 'Agg.', '$T = 4$', 'M' labels indicate the position of aggregates too large to enter the gel, peak of $T = 4$ capsids, and the monomer population. *Right*: the normalized intensity profiles obtained from the gel showing the spatial distribution and relative species population (sample – blue solid curve, monomer standard – grey dotted curve, $T = 4$ capsid population – dashed Gaussian red curve with shaded area). The total area under the blue curve is normalized to one, thus the area underneath the Gaussian equals the fraction of the total amount of material in the target $T = 4$ capsid and thus this area is equal to the yield.
